# Genomic Differentiation Along an Altitudinal–Longitudinal Gradient in Yarlung Zangbo Rock Agamas (*Laudakia*)

**DOI:** 10.3390/biology15211982

**Published:** 2026-11-02

**Authors:** Cong Wei, Jianchuan Li, Lifang Peng, Shiyang Weng

**Affiliations:** 1 Institute of Plateau Biology of Xizang Autonomous Region, Lhasa 850001, China; sws_weicong@sti.xizang.gov.cn; 2 Natural Science Museum of Xizang, Lhasa 850001, China; sws_jianchuan@sti.xizang.gov.cn; 3 State Key Laboratory of Plateau Ecology and Agriculture, Qinghai University, Xining 810016, China; lifang@qhu.edu.cn

**Keywords:** altitudinal gradient, genomic differentiation, genomic erosion, *Laudakia*, local adaptation, Qinghai–Tibet Plateau, whole-genome resequencing, Yarlung Zangbo

## Abstract

Extreme altitudinal gradients are natural experiments for testing how environmental gradients shape genomic variation, yet remain poorly characterised in high-altitude reptiles. We analysed whole-genome resequencing data (~17×) from 44 rock agamas (*Laudakia sacra* and *L. wui*) from five populations spanning 2311–3758 m in the Yarlung Zangbo valley, Qinghai–Tibet Plateau. Variation within *L. sacra* formed a continuous altitudinal cline rather than four discrete units, with the first principal component (PC1) explaining 41.29% of the variance; diversity declined monotonically with altitude (π fell ~9-fold), linkage disequilibrium (LD) half-decay lengthened 27-fold and the inbreeding coefficient (FROH) reached 0.155 with effective population size lowest at the valley head. Redundancy analysis with permutation identified 593 altitude-associated SNPs, jointly supported by a rank-based cline screen (6.1-fold enrichment over independence), and selective sweep analysis detected 4573 candidate genes across the ten pairwise comparisons, with the extreme interspecific contrast (C1 vs. C5) spanning cytoskeletal, epigenetic, glycolytic and oxygen-sensing functions. Low diversity, high inbreeding, small effective size and large projected genetic offset coincided at the valley head, making it the most genetically depauperate and climate-vulnerable population. These results reveal genomic differentiation along the altitudinal–longitudinal gradient consistent with putative incipient local adaptation and identify conservation priorities for high-altitude reptiles.

## Simple Summary

High mountains create steep environmental gradients, and the animals living along them must cope with very different conditions over short distances. We studied rock agamas (a group of lizards) living along the Yarlung Zangbo River valley on the Qinghai–Tibet Plateau, from warmer lowlands to cold high-altitude headwaters. By sequencing the genomes of 44 lizards, we found that populations living higher up differ genetically in a way that follows the altitude gradient rather than falling into separate groups. Lizards at the highest site have the least genetic diversity, the highest inbreeding and the smallest population size. Our models also indicate the highest projected genetic offset under future warming, suggesting potentially elevated genomic vulnerability. This combination makes the headwater population especially vulnerable. Our study shows how altitude shapes the genetic make-up of mountain animals and highlights where future conservation efforts should focus.

## 1. Introduction

The Qinghai–Tibet Plateau, the largest and highest plateau on Earth, harbours exceptionally rich biodiversity and is recognised as one of the world’s biodiversity hotspots, owing to its complex topography, steep altitudinal gradients and distinctive climate [1,2]. The sustained uplift of the Plateau and Quaternary climatic oscillations have profoundly shaped the distribution patterns and evolutionary trajectories of regional species [3,4]. The Yarlung Zangbo River, the most important river system in the southern Plateau, runs west to east across the southern margin of the Plateau and forms a continuous ecological gradient from alpine desert to subtropical gorges [5], providing a natural laboratory for studying altitudinal adaptive differentiation and speciation [3].

The genus *Laudakia* Gray, 1845 [6] (Agamidae) comprises about 20 species widely distributed across arid rocky habitats from Greece and the Middle East through Central Asia to the Gobi-Altai and the Yarlung Zangbo River Valley [7,8]. Six species are known from China, of which four (*L. sacra*, *L. wui*, *L. tuberculata* and *L. papenfussi*) are endemic to Xizang and are assigned to the Yarlung Zangbo group (*L. sacra* + *L. wui*) and the Himalaya group (*L. tuberculata* + *L. papenfussi*) [9,10]. *L. sacra* (Smith, 1935) [11] mainly inhabits the relatively high-altitude arid valleys of the middle-to-upper Yarlung Zangbo River, whereas *Laudakia wui* (Zhao, 1998) is endemic to the tropical and subtropical monsoon rainforest gorges of the lower Yarlung Zangbo River, distributed from Pailong through Tongmai to Mêdog County at elevations of approximately 700–2300 m [12,13,14]. The marked differences between the two species in distribution, altitudinal niche and phenotype suggest distinct evolutionary histories [12].

Recent years have seen important progress in *Laudakia* genomics. Yan et al. [15] assembled the first reference genome of *L. sacra* (1.80 Gb, N50 = 20.19 Mb), identifying key high-altitude adaptation genes such as *HIF1A*, *TIE2*, *ERCC6* and *MSH2*, and detecting a convergent evolution signal at the *MLIP* gene shared between *L. sacra* and the Tibetan hot-spring snake (*Thermophis baileyi* [16]). Chromosome-scale genomes of *L. wui* have subsequently been published: Wei et al. [13] assembled the first chromosome-level genome, and Tan et al. [14] further constructed a high-quality reference genome for its low-altitude Mêdog population, addressing its adaptation to the low-altitude hot-humid environment. However, population genomic studies of the genus lag far behind, and its population structure, mechanisms of high-altitude adaptation and speciation processes remain unresolved at the whole-genome scale.

The integration of whole-genome resequencing (WGR) with population genomic approaches provides unprecedented resolution for resolving the evolutionary processes of non-model organisms [17,18,19]. Analysis of genome-wide single-nucleotide polymorphism (SNP) data enables precise reconstruction of population structure, inference of historical demography, detection of gene flow events and localisation of adaptively differentiated genomic regions through selective sweep analysis [20,21]. Here, we performed WGR of 44 individuals from five geographic populations of *L. sacra* and *L. wui* along the Yarlung Zangbo River Valley to test two hypotheses: (i) that genomic variation is organised along the altitudinal–longitudinal gradient of the valley as a continuum rather than as discrete units and (ii) that divergence along this gradient is shallow relative to the species boundary, with intraspecific altitudinal differentiation weaker than the species-level divergence between *L. sacra* and *L. wui*. Altitude and longitude vary together along this transect (Pearson r = −0.95, Spearman ρ = −1.00); therefore, we treat them as a single composite gradient and frame the hypotheses in terms of position along that gradient; whether the environmental and geographic components can, nevertheless, be partially separated is addressed in Section 3.2 and Section 4.1. We predicted that high-altitude populations would exhibit reduced genetic diversity and elevated genomic inbreeding relative to mid-altitude populations. This prediction follows from the contrasting histories of the two axes. The four conspecific populations are recently derived from a common ancestor and occupy a continuously connected valley, so their differentiation is expected to remain modest and to track the environmental gradient rather than to accumulate into discrete units. By contrast, the species boundary reflects a longer and deeper separation between species with different altitudinal niches, and therefore, a much larger genomic divergence.

This study provides a population-genomic transect of a reptile along the steep altitudinal gradient of the Yarlung Zangbo valley, using it as a natural experiment to examine how environmental gradients are associated with local adaptation and genomic differentiation. Molecular evidence for such gradients has come almost entirely from fishes [22,23], while genome-wide work in lizards [24,25] has not yet examined a single continuously sampled river-valley gradient. Using genome-wide data, we ask how this composite gradient is associated with genomic divergence relative to the species boundary, and we reconstruct the demographic history of the sampled populations and project their genomic vulnerability under future climate, providing a baseline for conservation prioritisation in high-altitude reptiles.

## 2. Materials and Methods

### 2.1. Sample Collection

**Figure 1:**
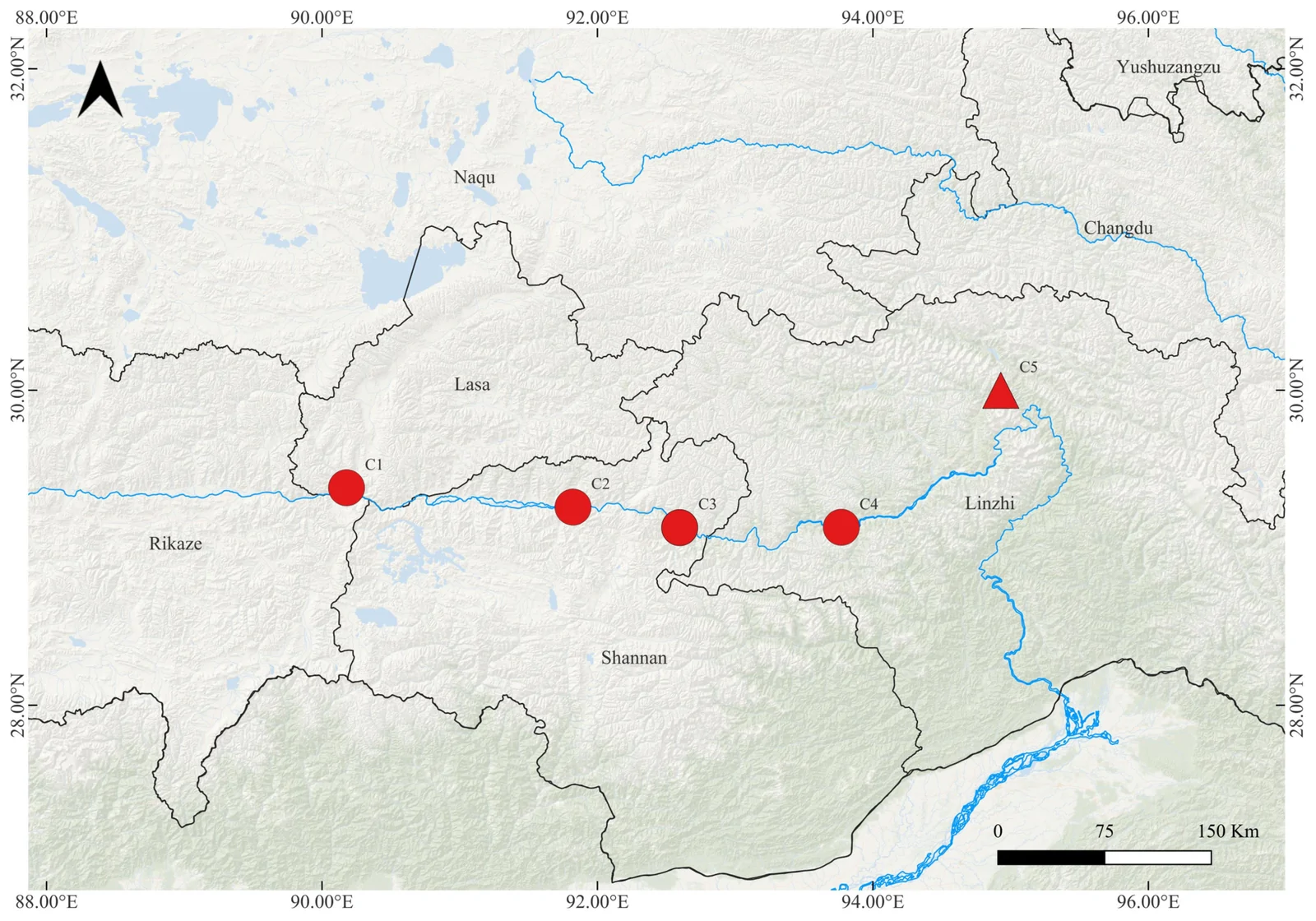
Sampling sites of five *Laudakia* populations along the Yarlung Zangbo valley (WGS 84 geographic coordinates, 28–32° N, 88–96° E; scale bar 0–150 km shown). Red circles: *L. sacra* (C1–C4); red triangle: *L. wui* (C5).

All animal procedures were approved by the Institutional Review Board of the Institute of Plateau Biology of Xizang Autonomous Region (protocol code LL20250003, approved on 16 July 2025), and field sampling was conducted under permit Zanglinhuxuzhun (2025) No. 11 issued by the Forestry and Grassland Administration of Xizang Autonomous Region. Five populations of *L. sacra* (C1–C4) and *L. wui* (C5) were sampled from west to east along the Yarlung Zangbo River in September 2025 (*n* = 44): C1 (*n* = 9, Nyemo, 29.3849° N, 90.1776° E, 3758 m), C2 (*n* = 9, Zetang Bridge, 29.2624° N, 91.8221° E, 3582 m), C3 (*n* = 10, Gyaca, 29.1323° N, 92.5965° E, 3265 m), C4 (*n* = 8, Mainling, 29.1345° N, 93.7704° E, 3017 m) and C5 (*n* = 8, Layue Hot Spring, 29.9994° N, 94.9282° E, 2311 m) (Figure 1). *L. wui* ranges from Pailong through Tongmai to Mêdog along the lower Parlung Tsangpo and Yarlung Zangbo gorges; Layue Hot Spring is the westernmost locality sampled in this study and lies at the upper altitudinal limit of the species, so it should not be read as representative of the species’ typical low-altitude habitat. Individuals were captured by hand and euthanised with an overdose of sodium pentobarbital delivered by intraperitoneal injection; death was confirmed before tissue collection, and no anaesthesia beyond the lethal dose or additional analgesia was required. Euthanasia was performed to allow the collection of fresh gut contents and gut microbiome samples for a companion study; muscle tissue for the present whole-genome resequencing was obtained from the same individuals so that no additional animals were sacrificed solely for this study and the total number of animals used across the two projects was minimised. The animals were never held in captivity, so housing and husbandry conditions do not apply. Muscle tissue was preserved in 95% ethanol, and tissue samples together with the corresponding voucher specimens were deposited in the collection of the Institute of Plateau Biology of Xizang Autonomous Region (Lhasa, China). Along this ~5° longitude transect, elevation falls monotonically (3758 → 2311 m) and co-varies with other climatic variables (PCA of nine environmental variables; Figure S15), so we used elevation as the primary environmental axis. This study is reported in accordance with the ARRIVE guidelines 2.0 (https://arriveguidelines.org).

### 2.2. DNA Extraction and Whole-Genome Resequencing

Genomic DNA was extracted from muscle tissue using a standard phenol–chloroform protocol. After quality control, 1 μg of genomic DNA was randomly fragmented with a Covaris sonicator (Covaris, Inc., Woburn, MA, USA), and fragments of 200–400 bp were selected using MGIEasy DNA Clean Beads (CAT#1000005279, MGI Tech Co., Ltd., Shenzhen, China). Selected fragments were end-repaired, 3′-adenylated, adapter-ligated and PCR-amplified, and the products were purified with MGIEasy DNA Clean Beads. Double-stranded PCR products were heat-denatured and circularised into single-stranded circular DNA (ssCir DNA) libraries using the MGIEasy circularisation module (CAT#1000005260, MGI Tech Co., Ltd., Shenzhen, China) via splint oligonucleotides. Qualified libraries were paired-end sequenced (PE150) on the DNBSEQ-T7RS platform (MGI Tech Co., Ltd., Shenzhen, China). Raw reads were filtered with fastp v0.23.2 to remove adapters and low-quality bases [26], yielding 1357.18 Gb of clean data (mean 30.85 Gb per sample), with Q20 > 98.7% and Q30 > 96.0%. The mean duplication rate was 1.4% (range 1.2–1.8% across the 44 libraries), and the insert-size distribution peaked at 256 bp, with a median of 214 bp. Clean reads were aligned to the *L. sacra* reference genome (GWH Assembly ID: GWHBKHB00000000; 1.80 Gb, N50 = 20.19 Mb) [15] using BWA-MEM v0.7.17 [27], with a mean mapping rate of 99.87%, mean depth of ~17× and mean genome coverage of 98.7%. Raw sequencing data have been deposited in the NCBI Sequence Read Archive under BioProject PRJNA1524104 (SRA study SRP734744). Variants were called with GATK v4.2 HaplotypeCaller [28]; after VQSR quality filtering and population filtering (maf 0.05, min-meanDP 5, max-missing 0.75), 21,911,886 high-quality SNPs and 2,651,703 InDels were retained. Variants were functionally annotated with ANNOVAR (version 20191024) [29]. Downstream analyses used nested subsets of this call set: 594,884 LD-pruned SNPs for the population structure, phylogenetic, PCA and ADMIXTURE analyses (Section 2.3); a 198,685-SNP matrix (missing < 30% within C1–C4, MAF > 5%) for the genotype–environment association analysis (Section 2.7), a 198,742-SNP matrix (missing < 30% across all individuals, MAF > 5%) for the interspecific divergence analysis (Section 2.9) and 3725 SNPs within candidate-gene intervals for anchoring candidate genes to altitudinal clines (Section 2.8).

### 2.3. Population Structure Analysis

The SNP dataset was LD-pruned with PLINK v1.90 (--indep-pairwise 50 10 0.1) [30], retaining 594,884 LD-independent SNPs for population structure analysis. A maximum-likelihood phylogenetic tree was constructed with IQ-TREE v2.0 under the GTR model with 1000 ultrafast bootstrap (UFBoot) replicates [31]. Principal component analysis (PCA) was performed with VCF2PCACluster v1.40 [32]. Population genetic structure was inferred with ADMIXTURE v1.3 for K = 1–10, and the optimal K was determined by cross-validation error [33]. Pairwise Wright’s fixation index (FST) was computed with VCFtools v0.1.16 [34].

### 2.4. Genetic Diversity Analysis

Sliding-window analyses (100 kb) were performed for each population with VCFtools [34] to compute nucleotide diversity (π), observed heterozygosity (Ho), expected heterozygosity (He), inbreeding coefficient (FIS) and Tajima’s D. These statistics were computed on the same MAF-filtered variant set used for the remaining analyses; because a minor-allele-frequency threshold truncates the low-frequency end of the site-frequency spectrum, the resulting π and Tajima’s D are not directly comparable with estimates obtained from unfiltered data, and the values of FIS are likewise conditional on that filtering. Therefore, Tajima’s D is interpreted descriptively rather than as a test of departure from neutrality. To quantify the effect of that filtering, we recomputed π, Tajima’s D and FIS in parallel from the unfiltered call set (biallelic SNPs passing the same hard filters and a per-population call rate of 0.8, but with no minor-allele-frequency threshold); the two site sets differ only in the allele frequency filter, so any difference between them is attributable to that filter alone. We tested whether pairwise differentiation increases with position along the transect using rank-based (Spearman) Mantel tests between the matrix of pairwise FST among the four *L. sacra* populations and two explanatory distance matrices: absolute altitudinal difference and great-circle geographic distance computed from the sampling coordinates (Section 2.1). Because the two explanatory matrices are themselves correlated, we additionally ran partial Mantel tests, in which each explanatory matrix was regressed on the other and the Mantel test was applied to the residuals, so that the effect of one axis could be assessed while controlling for the other. All tests used 200,000 permutations and were repeated for the full set of ten population pairs, including C5. Genome-wide linkage disequilibrium (LD) decay was analysed with PopLDdecay v3.41 [35], recording the physical distance at which the LD coefficient (r^2^) decayed to half of its maximum (half-decay distance). To test the relationship between genetic diversity indices and altitude, Spearman rank correlation and ordinary least-squares linear regression were performed between population means and altitude for the four *L. sacra* populations (C1–C4); *L. wui* (C5) was treated as a separate species and plotted but excluded from the regression. Statistical analyses were conducted in R v4.3.

### 2.5. Gene Flow Analysis

Population divergence history and gene flow events were inferred with TreeMix v1.13 [36]. Allele frequency data were used as input, with block size (-k) set to 1, 500 and 1000, and migration edges (-m) from 0 to 10, each with 10 replicates. The optimal number of migration edges was determined with the OptM R package based on the Evanno method [37]. The results with k = 1000 and m = 1 were taken as the best model.

### 2.6. Selective Sweep Analysis

A comprehensive selective sweep analysis was conducted to detect genomic regions [38] of adaptive differentiation between populations. Window-based FST (100 kb windows, 10 kb step) was computed for each population pair, together with nucleotide diversity (π) for each population, from which between-population π ratios were derived. Regions in the intersection of the top 5% FST and top 5% π-ratio thresholds were defined as candidate selective sweep regions [38]. Genes within candidate regions were subjected to GO and KEGG enrichment analyses using Fisher’s exact test, with *p*.adjust < 0.05 as the significance threshold. Particular attention was paid to the extreme altitudinal contrast between C1 (highest) and C5 (lowest) to identify candidate genes related to altitudinal adaptation.

### 2.7. Genotype–Environment Association Analysis

We subsampled 198,685 SNPs (missing < 30% within C1–C4 and MAF > 5%; this is a slightly smaller set than the 198,742 SNPs used for the interspecific divergence analysis in Section 2.9, where missingness is assessed across all five populations) and computed the Spearman correlation between population allele frequency and altitude across the four *L. sacra* populations (C1–C4; C5 excluded as a distinct species to avoid cross-species mapping bias). *p*-values were Benjamini–Hochberg-adjusted [39], and SNPs with |ρ| ≥ 0.9 were retained as altitude-associated candidates; as explained in Section 3.6, this threshold is descriptive rather than inferential with only four populations, and the statistical support for the resulting set comes from the permutation test described below. To cross-validate, we ran a redundancy analysis (RDA) on the individual genotypes (0/1/2 dosage) of the 36 *L. sacra* individuals against standardised altitude (scale = FALSE; [40]), imputing missing genotypes with the locus mean.

Two design features shape inference. First, the four *L. sacra* populations are ordered along the valley, so an allele frequency cline cannot separate altitudinal selection from spatial autocorrelation; we therefore treat the cline screen as a descriptive filter and base inference on the RDA. Second, individuals within a population are not exchangeable, so permuting individual altitude labels would be anticonservative; we instead permuted population altitude labels (all 4! = 24 permutations, enumerated exhaustively) and report exact *p*-values. The 593 SNPs supported jointly by both methods are our primary candidate set.

### 2.8. Candidate-Gene Extraction and Cline Analysis

To anchor the candidate genes identified by selective sweep analysis to altitudinal selection, we extracted all SNPs within the genomic intervals of 13 candidate genes—*FLNC* (scaffold 30: 14,156,172–14,245,297), *SHROOM4* (scaffold 26: 547,525–573,237), *HDAC8* (scaffold 26: 498,773–504,572), *DGKD* (scaffold 26: 582,473–614,213), *BMP15* (scaffold 26: 540,049–543,238), *Eno3*, *PKM*, *HK2*, *CYBA*, *Piezo1*, *ARRB2*, *MTHFD2* and *SLC4A5*—from the filtered VCF (3725 SNPs in total). These 13 genes were selected from the selective sweep candidates on the basis of their functional annotations relevant to high-altitude physiology (cytoskeletal remodelling, epigenetic regulation, signal transduction, glycolysis, oxygen sensing, one-carbon metabolism and GPCR signalling) and their altitudinal allele frequency clines, which were stronger than the genome-wide background. For each SNP, we calculated the population allele frequency and the Spearman correlation with altitude across the four *L. sacra* populations (C5 was excluded as a distinct species). We then compared the distribution of |ρ| for the candidate-gene SNPs to the genome-wide background (198,685 SNPs from the same filtered VCF) using the Mann–Whitney U test. Genomic intervals are given in the text for the five genes with resolved coordinates; the remaining eight genes were anchored using their annotated gene models in the same filtered VCF.

### 2.9. Interspecific Divergence

We quantified the depth of divergence between *L. wui* (C5) and *L. sacra* (C1–C4) from a 198,742-SNP matrix (missing < 30% across all individuals and MAF > 5%; this is slightly larger than the 198,685 SNPs used for the GEA in Section 2.7 because missingness is assessed across all five populations rather than within C1–C4) using three summary statistics: dXY (the probability that two alleles drawn at random from the two groups differ, computed as *p*_1_(1 − *p*_2_) + *p*_2_(1 − *p*_1_) and averaged over all SNPs); the proportion of fixed-difference SNPs (allele frequency < 0.05 in one group and > 0.95 in the other); and Hudson’s FST [41]. dXY is an absolute measure of divergence between two groups; it is not bounded above by their within-group diversity and is not a per-site nucleotide divergence. A 0.05/0.95 threshold was used for fixed differences to accommodate residual genotyping error and low-frequency polymorphism at the sampled coverage. Each statistic was compared between interspecific (C5 vs. C1–C4) and intraspecific (C1–C4 pairwise) comparisons. To further separate the altitudinal and species axes of divergence, dXY was additionally computed between the high-altitude (C1 + C2) and low-altitude (C3 + C4) *L. sacra* groups (altitudinal axis) and between C5 and the low-altitude *L. sacra* group (species axis).

### 2.10. Genetic Offset Under Future Climate

Genomic vulnerability to climate change was estimated by projecting RDA allele-frequency variation onto the current altitudinal gradient (altitude and longitude as predictors) and predicting allele frequencies under the Shared Socioeconomic Pathway 5–8.5 (SSP585) scenario for 2071–2100 [42]. Following elevation-dependent warming [43], future temperature was modelled as an equivalent downward shift in altitude (4.5–6.0 °C ≈ 692–923 m). Genetic offset is the Euclidean distance between current and projected RDA scores. With only four *L. sacra* populations, we used linear RDA projection, so offsets are relative rankings rather than absolute predictions. In addition, the projected shift of 692–923 m lies below the altitudinal range sampled within *L. sacra* (3017–3758 m), so the projection extrapolates beyond the calibration range; the offsets should therefore be compared between populations rather than read as predicted values. As a cross-check, we computed the risk of non-adaptedness (RONA; [44]).

### 2.11. Demographic History Reconstruction

Historical effective population size (Ne) trajectories were reconstructed for each population with SMC++ v1.15 (based on the sequentially Markovian coalescent; [45]). Ne histories were converted to calendar years assuming a mutation rate of 1.17 × 10^−8^ per site per generation and a generation time of 3 years [46]. Because the most recent and deepest epochs are weakly constrained, inference focused on intermediate epochs and on the relative ordering of populations rather than absolute ages. As an independent estimate of contemporary effective size, we applied the linkage-disequilibrium method, fitting r^2^(d) = 1/(1 + 4·Ne·c) + 1/S to the observed LD decay curves, where c is the recombination rate (assumed to be 1 cM/Mb) and S the number of gene copies sampled; because the absolute values scale with the assumed recombination rate, only the relative ordering between populations is interpreted.

### 2.12. Runs of Homozygosity and Genomic Inbreeding

Genomic inbreeding was characterised from runs of homozygosity (ROHs). ROHs were identified for each individual with PLINK v1.90 [30] on the full quality-filtered SNP set (all VQSR-passing SNPs; i.e., without the MAF filter used for the genotype–environment analysis), using a sliding window of 50 SNPs (--homozyg-window-snp 50), allowing three heterozygous and five missing sites per window, with a minimum ROH length of 500 kb. ROH were classified into length bins (0.5–1, 1–2 and 2–4 Mb), and the genomic inbreeding coefficient FROH was calculated for each individual as the total length of ROH divided by the autosomal genome length (1,797,983,225 bp). Population FROH distributions were summarised and compared between the five populations. Best practices for ROH detection and interpretation in wild populations follow [47].

### 2.13. Mutational Load Analysis

To quantify the relative burden of deleterious mutations, we classified SNPs from the filtered VCF using ANNOVAR together with the gene models and annotation files supplied with the sequencing service. SNPs were categorised as nonsynonymous (*n* = 89,100) or synonymous (*n* = 133,959); the remaining 1056 exonic SNPs (stop-gain/stop-loss and related classes) fall outside these two categories. For each individual, we computed the minor-allele load at nonsynonymous and synonymous sites separately and expressed the relative load as the nonsynonymous-to-synonymous ratio, which reflects the efficiency of purifying selection [48]. The *L. wui* population (C5) was excluded because cross-species annotation biases would confound load estimates.

### 2.14. Use of Artificial Intelligence Tools

Artificial intelligence (AI) tools were used for language polishing and structural editing of this manuscript. The authors reviewed, edited, and take full responsibility for all content. No AI tool is listed as an author.

## 3. Results

### 3.1. Sequencing Data and Variant Calling

We obtained 1357.18 Gb of clean data for the 44 individuals (mean 30.85 Gb, range 25.70–37.81 Gb; Q30 > 96.0%). After alignment to the reference, the mean mapping rate was 99.87% and mean depth was ~17× (mean genome coverage 98.7%). Stringent filtering retained 21,911,886 SNPs and 2,651,703 InDels, of which 224,115 SNPs (1.02%) were exonic (89,100 non-synonymous). After LD pruning, 594,884 SNPs were retained for population structure analysis.

### 3.2. Population Genetic Structure and Species Divergence

The IQ-TREE phylogeny (Figure S1) was strongly supported (most nodes bootstrap ≈ 100%). C1 and C2 each formed a monophyletic clade; within the eastern clade, C5 (*L. wui*) formed a long, fully supported branch (bootstrap = 100%) sister to C4, indicating shared ancestry with the eastern *L. sacra* populations. The deepest split separated the high-altitude west (C1/C2) from the eastern clade (C3–C5), matching the west–east geographic order; the sister relationship of C5 to C4 is compatible with derivation from a mid-to-low-altitude ancestor shared by both species (Section 4.2).

**Figure 2:**
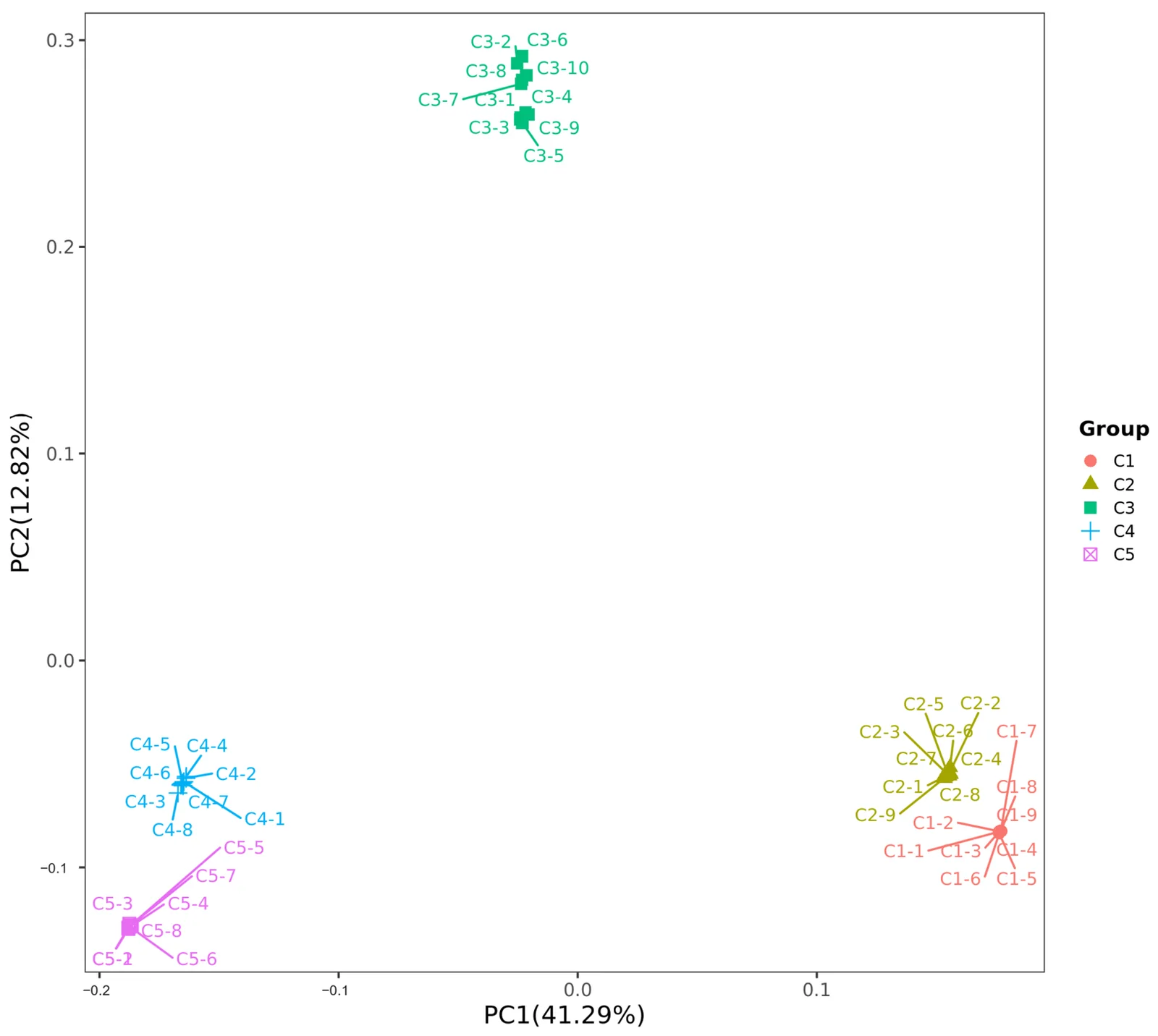
Principal component analysis (PC1 vs. PC2) of the five *Laudakia* populations from the Yarlung Zangbo valley, based on LD-pruned SNPs. Populations C1–C5 are distinguished by colour and point shape, as indicated in the legend.

PCA (Figure 2) resolved a continuous west–east gradient (C1 → C2 → C3 → C4 → C5): PC1 (41.29%) ordered the populations along the transect, PC2 (12.82%) separated C3, and PC3 (7.37%) separated C4 from C5 (together 61.48% of variance). Because C5 is the only sampled *L. wui* population, the species axis cannot be separated from the population axis in the PCA, so we interpret the gradient primarily within *L. sacra*.

**Figure 3:**
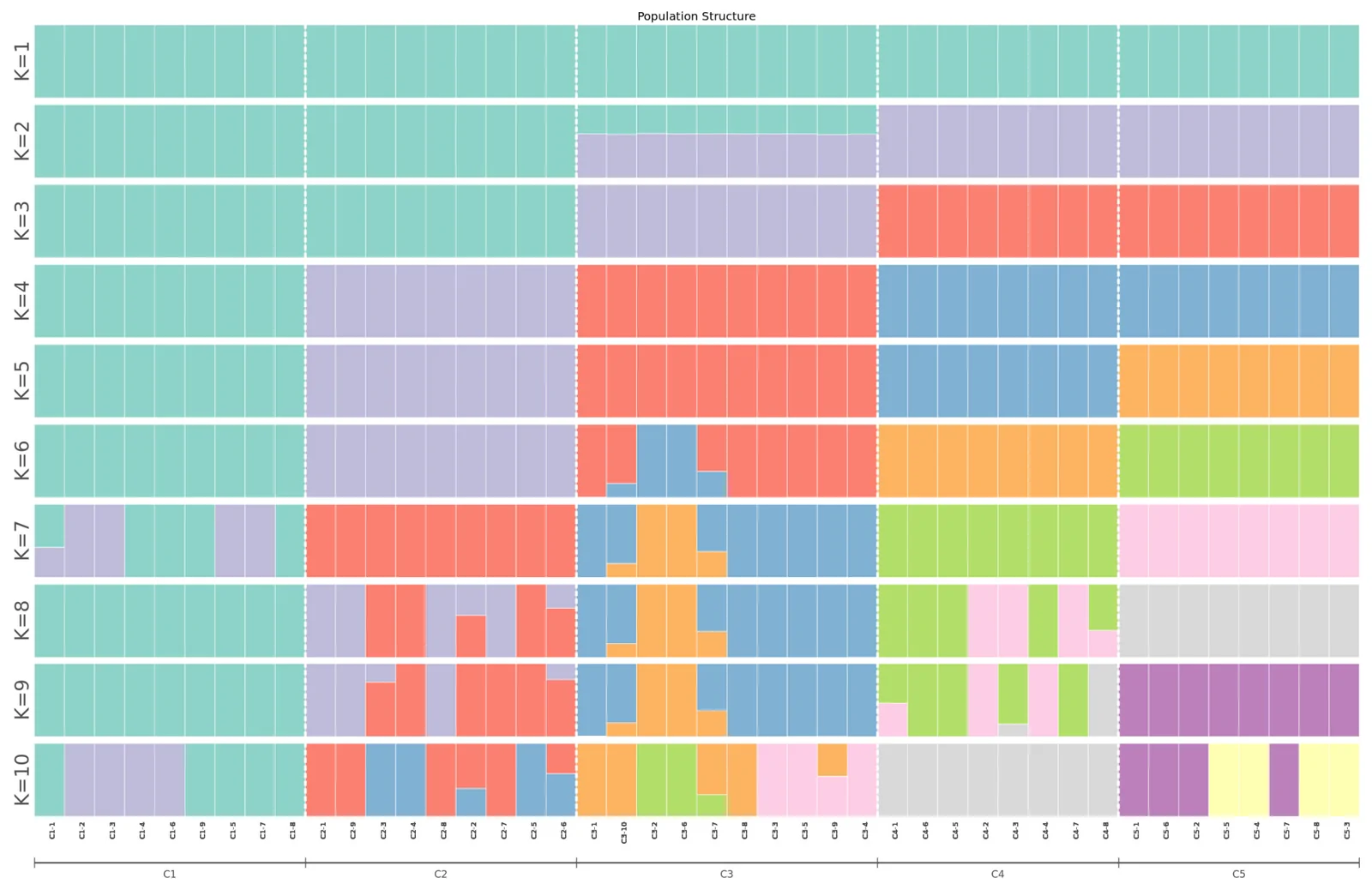
ADMIXTURE ancestry proportions for K = 1 to K = 10 for the five *Laudakia* populations from the Yarlung Zangbo valley (the optimal K = 5 was selected using the minimum cross-validation error). Each vertical bar represents one individual; colours denote inferred ancestral components.

ADMIXTURE (Figure 3) recovered multi-level structure: cross-validation error was minimal at K = 5 (0.386; Figure S5), matching the five populations. The K = 2 solution separated high-altitude (C1/C2) from mid-to-low-altitude (C3/C4/C5) populations; because ADMIXTURE components are not ordered in time, we describe this as the strongest axis of structure rather than the earliest split. Within *L. sacra*, this axis tracks altitude, and C5 emerged as a distinct cluster only at K = 5. Because *L. wui* is represented by a single population, the species and population axes cannot be separated at any K, so the low-K grouping of C5 with C3/C4 is not interpreted as evidence about species boundaries. At K = 5, each population was nearly homogeneous (Q > 0.99), indicating very limited recent gene flow.

Pairwise FST (Table S2; Figure S6) was highest between C1 and C5 (0.898); all C5 comparisons exceeded 0.80 (0.806–0.898), far higher than all intraspecific comparisons (0.374–0.675), supporting the species status of *L. wui*. Within *L. sacra*, FST increased with position along the transect (analysed in detail at the end of this section).

Interspecific divergence substantially exceeded intraspecific altitudinal differentiation (Figure S18). Across 198,742 SNPs, dXY averaged 0.646 between C5 and C1–C4 versus 0.136 within *L. sacra* (4.8-fold); fixed differences were 35.0% versus ≈2%; and Hudson’s FST was 0.754 versus 0.398. The apparent magnitude of the species–altitudinal contrast therefore depends on the statistic used: dXY gives 4.8-fold and fixed differences 17.5-fold, whereas FST gives only 1.9-fold, because FST is bounded above and saturates as allele frequencies approach fixation. Decomposing by axis, altitudinal differentiation within *L. sacra* was 0.142 (high- vs. low-altitude groups) versus 0.639 between *L. wui* and low-altitude *L. sacra* (4.5-fold), showing that intraspecific altitudinal divergence remains at a shallow, pre-completed stage relative to the species boundary. All reads were mapped to a single *L. sacra* reference, which may inflate divergence estimates involving *L. wui* relative to an analysis based on two species-specific references, so the magnitudes reported here should be treated as upper bounds for the interspecific contrast. Within *L. sacra*, differentiation increased with position along the transect, and the two explanatory axes behaved differently. A Mantel test between pairwise FST and absolute altitudinal difference gave r = 0.943 (200,000 permutations, *p* = 0.0166), whereas the same test against geographic distance gave r = 0.771 (*p* = 0.104). Because the two explanatory matrices are correlated (Pearson r = 0.83), we also ran partial Mantel tests: with geographic distance controlled, altitudinal difference remained significant (partial r = 0.943, *p* = 0.0166), whereas with altitude controlled, geographic distance explained nothing (partial r = 0.086, *p* = 0.919). The same contrast held across all ten population pairs, including C5 (Mantel r = 0.976, *p* < 0.0001 for altitudinal difference and r = 0.745, *p* = 0.0176 for geographic distance; partial r = 0.879, *p* = 0.0016 and r = −0.345, *p* = 0.331, respectively). Within the limits of six (or ten) population pairs, these results favour an environmental-gradient interpretation over pure isolation by distance, although altitude is itself a composite proxy for several collinear climatic variables and no single driver can be identified from these data.

### 3.3. Genetic Diversity and Evolutionary Dynamics

Genetic diversity is summarised in Table 1 (distributions in Figure S7). Within *L. sacra*, π declined monotonically with altitude (Figure S8), from 0.0013 in C4 (3017 m) to 0.00015 in C1 (3758 m) (Spearman ρ = −1.00; linear r = −0.99, *p* < 0.05, R^2^ = 0.97); the SNP density and He showed the same trend. As a sister species, *L. wui* (C5, 2311 m) had the highest π (0.0015), He (0.404) and SNP density, but this reflects long-term variation accumulated within an independent species rather than a simple altitude effect. Tajima’s D was negative in C1 (−0.303, excess low-frequency alleles) and positive in C2–C5 (0.731–1.206); given the MAF filtering described in Section 2.4, these values are reported for completeness and are not used below to infer the direction of demographic change.

LD decay differed markedly between populations (Table S3; curves in Figure S9): half-decay distance was shortest in C3 (2.4 kb) and C4 (2.9 kb) but longest in C1 (65.3 kb) and C2 (42.4 kb), reflecting much smaller effective population sizes at high altitude; C5 was intermediate (11.5 kb).

### 3.4. Gene Flow and Population Evolutionary Relationships

TreeMix (Figure S2) recovered the topology (C2, ((C4, (C5, C3)), C1)), in which C5 was sister to C3 rather than to C4 as in the IQ-TREE tree, and one migration edge with weight 0.444 connecting the C1 lineage to the ancestor of C3. Because the direction of TreeMix migration edges is generally not identifiable, this is best read as evidence of historical gene flow between the C1 and C3 lineages rather than as a statement about donor and recipient, and the edge was selected by OptM without a formal bootstrap test. The residual heatmap showed no systematic bias (Figure S10), indicating a good fit; recent gene flow between populations was very limited.

### 3.5. Selective Sweeps and Candidate Genes

Joint FST and π-ratio sweeps identified 4573 candidate gene hits across the 10 pairwise comparisons (non-deduplicated). The per-comparison counts did not simply track pairwise divergence. The largest set came from C3 vs. C4 (877 genes), where the two populations retain comparable diversity yet exchange little, whereas the extreme interspecific contrast C1 vs. C5 yielded only 112 and the genetically closest pair C1 vs. C2 yielded 100. This is because the joint screen requires high FST and a one-sided reduction in diversity, a combination that becomes rare once divergence is genome-wide. The 112 C1 vs. C5 candidates were enriched for cytoskeletal functions (Figures S11–S13).

GO enrichment (*p*.adjust < 0.05) of the C1 vs. C5 candidates was significant only for cytoskeleton-related functions (actin filament organisation, actin filament binding and calmodulin binding). Other comparisons were enriched for olfactory receptor activity (C1 vs. C3), chemokine and microfilament motor activity (C1 vs. C4), and wound healing and monosaccharide transport (C3 vs. C4); carbonate dehydratase activity in C2 vs. C3 was marginal (*p*.adjust = 0.055).

KEGG enrichment was weaker than GO; only olfactory transduction and nicotine addiction reached significance (C1 vs. C3, *p*.adjust < 0.01), likely reflecting limited KEGG annotation.

Several C1 vs. C5 candidates have known high-altitude functions: *SHROOM4* and *FLNC* (actin cytoskeleton and mechanotransduction), *HDAC8* (epigenetic regulation and hypoxia response), *DGKD* (lipid signalling and energy metabolism), *BMP15* (*TGF-β* pathway), *NR2E1* and *RPS4*. The cytoskeletal genes *FLNC* and *SHROOM4* recurred in the C1 vs. C5 and C2 vs. C5 comparisons (Figures S11–S13), suggesting a central role for cytoskeletal remodelling. Because C1 and C5 differ in altitude and species identity, these interspecific candidates cannot be attributed to altitude alone and are best read as candidates for altitudinal and species-level differentiation rather than as evidence of altitudinal adaptation.

### 3.6. Altitude-Associated Genomic Variation

**Figure 4:**
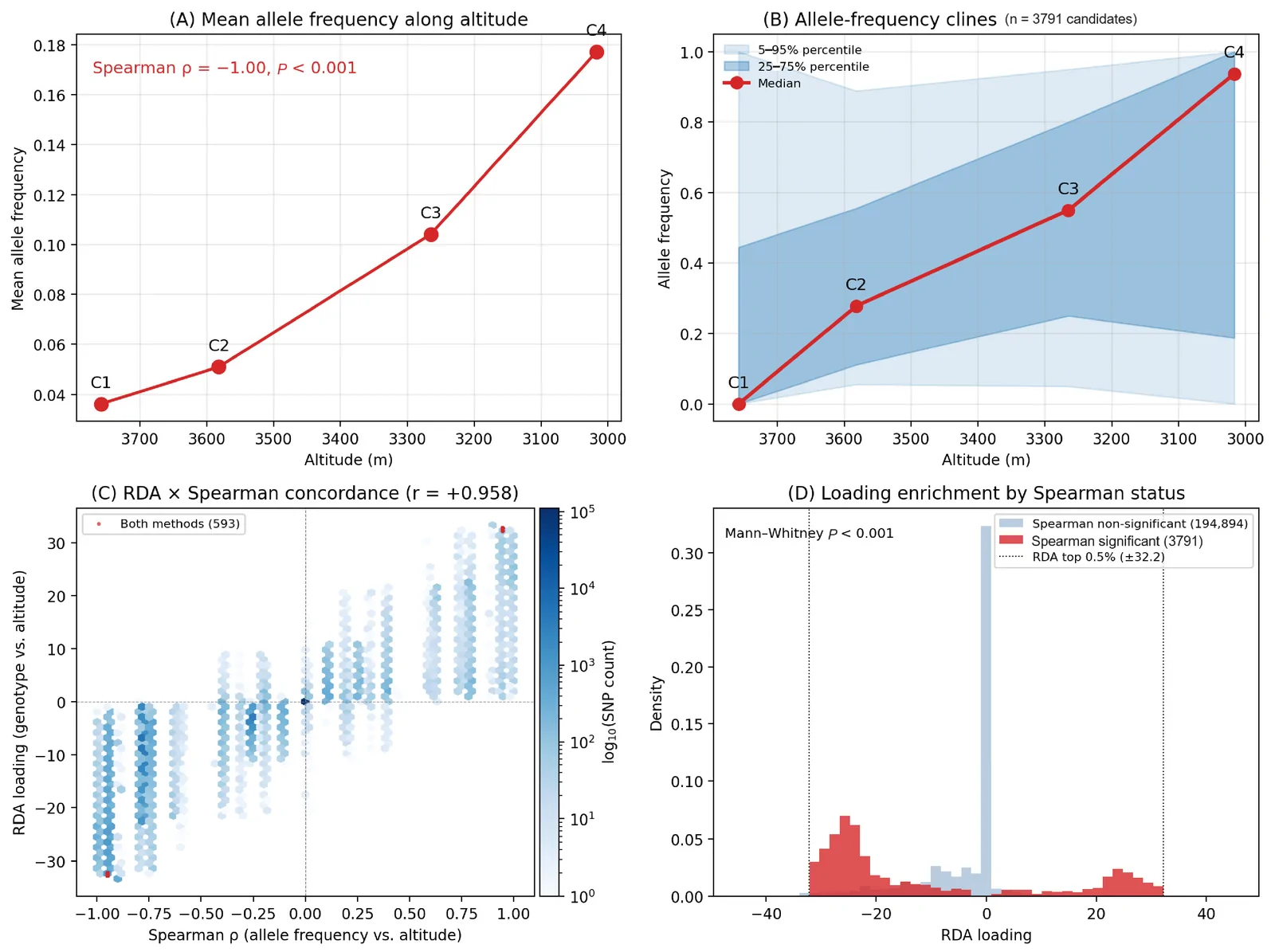
Genotype–environment association (GEA) with altitude across the four *L. sacra* populations (C1–C4) from the Yarlung Zangbo valley. (**A**) Mean allele frequency of the candidate SNPs declines monotonically with increasing altitude (Spearman ρ = −1.00 across *n* = 4 populations); because these SNPs were themselves selected based on this relationship, the panel illustrates the selection criterion rather than an independent result. (**B**) Allele frequency clines of the 3791 perfectly monotonic candidates (|ρ| = 1) returned by the descriptive first-pass screen; SNPs were oriented so that the allele that is most frequent at the low-altitude end of the transect is plotted first; the robust, cross-validated set is the 593 SNPs shown in (**C**); the red line shows the median and shaded bands the 25–75% and 5–95% percentiles. (**C**) Concordance between the two approaches: RDA loadings (individual genotypes vs. altitude) plotted against Spearman ρ; red points are SNPs jointly supported by both methods (*n* = 593). (**D**) Distribution of RDA loadings for Spearman-significant versus non-significant SNPs.

Mean allele frequency declined monotonically with altitude across the four *L. sacra* populations, from 0.177 in C4 to 0.036 in C1 (Figure 4A). A first-pass cline screen using |ρ| ≥ 0.9 returned 18,402 candidates (9.3% of the sites examined), of which 3791 (1.9%) changed perfectly monotonically across the four populations (Figure 4B); these were distributed across 171 of the 226 SNP-bearing scaffolds at close to the genome-wide expectation (Figure S16). This proportion must be read against the structure of the data. With only 9–10 individuals per population, allele frequencies are quantised on a coarse grid: among the biallelic sites scored in the four *L. sacra* populations, 56.6% showed an identical allele frequency across all four populations, and only 7.3% showed four distinct frequencies. Among those rankable sites, 26.0% were perfectly monotonic, 3.1 times the 8.33% expected by chance (2 of the 24 permutations). Because the smallest two-sided permutation *p* attainable with four populations is 2/24 = 0.083, no individual SNP passes a Benjamini–Hochberg FDR of 0.05 (the minimum attainable BH-FDR is ≈16,557); therefore, we treat this screen as a descriptive filter whose support comes from its joint behaviour with the RDA rather than from a per-SNP FDR. The 1049 SNPs with the largest RDA loading magnitudes (above the 99.5th percentile) overlapped the |ρ| ≥ 0.9 set at 593 SNPs—a 6.1-fold enrichment over the 97.2 expected under independence—whereas none of the 3791 perfectly monotonic (|ρ| = 1) SNPs was among them because SNPs with the largest altitudinal contrasts typically have tied population frequencies (for example, two-block patterns such as 1.0/1.0/0.0/0.0) that cap the rank correlation below one. RDA showed altitude explaining 39.4% of genotypic variation (RDA1), closely matching PC1 (41.29%); exhaustive permutation of population altitude labels (4! = 24) gave an exact *p* = 1/24 = 0.042, the smallest *p* attainable with four populations. The two approaches were highly concordant (RDA loadings vs. Spearman ρ: r = 0.958; sign agreement 99.1% genome-wide and 100% among the RDA-selected SNPs; elevated loadings, Mann–Whitney *p* < 0.001), so the 593 SNPs supported by both methods (RDA-loading outliers ∩ |ρ| ≥ 0.9; Figure 4C,D) are our robust altitudinal set.

Then, we asked whether the clines of these loci are gradual or stepped. For each perfectly monotonic SNP, we decomposed the total change in allele frequency into the three successive increments (C1–C2, C2–C3, and C3–C4) and computed a jump index, max|Δ|/Σ|Δ|, which is about one-third for a strictly linear cline before sampling noise is added. Across the 3791 perfectly monotonic SNPs, the mean jump index was 0.60, whereas a null generated from a strictly linear cline with the same endpoints and the same binomial sampling, subjected to the same monotonicity filter, gave 0.48 (*n* = 20,706 simulated loci; Kolmogorov–Smirnov statistic 0.41, *p* < 10^−300^). Therefore, allele-frequency clines at individual loci are steeper and more concentrated than a linear gradient, with a single interval accounting for about 60% of the total change on average. The largest increment was distributed across all three intervals (32.5%, 37.8% and 29.6%) and only slightly more concentrated than under the null (32.7%, 34.2% and 33.1%), so the data do not identify a single altitudinal threshold.

### 3.7. Anchoring Candidate Genes to Altitudinal Clines

We compared their altitudinal clines to the genome-wide background to test whether the sweep candidates are specific targets of altitudinal selection. Across 13 candidate genes (cytoskeleton, epigenetics, signal transduction, glycolysis, oxygen sensing, one-carbon metabolism, GPCR signalling), the mean |Spearman ρ| with altitude was 0.70 (0.51–0.76) versus 0.29 genome-wide (Figure S19); all 13 fell in the top quartile (Mann–Whitney *p* < 0.001). Because the candidate genes were identified using the same altitudinal gradient, this comparison is descriptive rather than independent. Four genes (*SHROOM4*, *HDAC8*, *BMP15*, *DGKD*) cluster in a ~115 kb interval on scaffold 26, which is consistent with—but does not demonstrate—a co-adapted region; establishing this would require haplotype and linkage-disequilibrium evidence for the interval. Eight additional high-altitude genes (*Eno3*, *PKM*, *HK2*, *CYBA*, *Piezo1*, *ARRB2*, *MTHFD2*, *SLC4A5*) also showed strong clines (|mean ρ| = 0.51–0.76), providing candidate loci for future functional study.

### 3.8. Genomic Vulnerability to Climate Change

**Figure 5:**
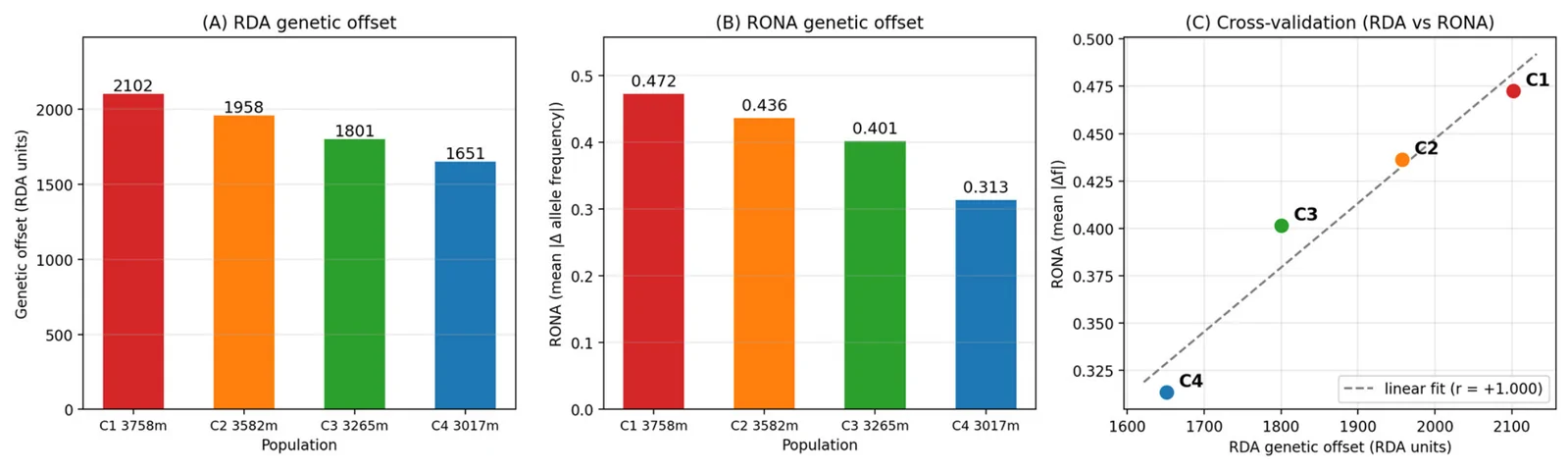
Genomic vulnerability of the Yarlung Zangbo *Laudakia* populations to climate change under the SSP585 scenario for 2071–2100. (**A**) RDA genetic offset for each population (RDA units). (**B**) RONA genetic offset for each population (mean |Δ allele frequency|). (**C**) Concordance between the RDA and RONA offsets (Spearman r = +1.000); because both are derived from the same projection onto a single gradient, this is a consistency check rather than an independent validation.

Genetic offset under SSP585 (2071–2100) increased monotonically with altitude, from 1651 RDA units in C4 to 2102 in C1 (Figure 5). C1, which also showed the lowest diversity and highest inbreeding, shows the largest projected genetic offset. RONA, computed from the same allele-frequency projections, recovered the same ordering (C1 > C2 > C3 > C4; Spearman r = +1.000; Figure 5C). Because RONA and the RDA offset are both derived from the same projection onto a single gradient, this concordance is a consistency check rather than an independent validation, and the two quantities should not be treated as separate lines of evidence.

### 3.9. Demographic History and Genomic Inbreeding

SMC++ (Figure S3) showed that the high-altitude C1 and C2 maintained persistently small effective sizes, declining to very small sizes (effective population size, C1 Ne ≈ 164; C2 ≈ 60), whereas C3 remained large (~10^4^) and the C4 trajectory was the least consistent among the four (see below). *L. wui* (C5) showed an ancient expansion followed by a mid-Holocene contraction. These trajectories are concordant with the LD-decay results, confirming that altitude is accompanied by a sustained reduction in effective population size. An independent estimate of contemporary effective size based on linkage disequilibrium gave a similar ordering of the extremes but different absolute values and a different position for C4 (C1 < C2 < C5 < C4 < C3 at an assumed recombination rate of 1 cM/Mb). Because these estimates scale with the assumed recombination rate, only the ordering should be compared with the coalescent results; at 3 cM/Mb the C1 estimate (≈147) approaches the SMC++ value (≈164). Two features of the comparison deserve note. First, C4 is the second-largest population by the LD method, consistent with its high π and low FROH and contrary to a small-population interpretation; therefore, several lines of evidence agree that C4 has not undergone a strong recent contraction. Second, the LD method places C1 below C2, whereas SMC++ places C2 below C1, so the relative ranking of the two high-altitude populations is not resolved.

ROH analysis (Figure S4; Table S1) independently confirmed this at the individual level. FROH rose steeply with altitude: C1 was by far the most inbred (mean 0.155, range 0.072–0.272; ~278 Mb in ROH), C2 intermediate (0.031), and C3/C4 an order of magnitude lower (0.006); C5 was least inbred (0.001). C1 carried not only the most abundant 0.5–1 Mb segments but also the longest (2–4 Mb) ROH (Figure S14), signalling recent inbreeding and small recent Ne. Nevertheless, the same populations showed a genome-wide excess of heterozygotes rather than a deficit: FIS was negative in C1 (−0.181; 95% CI −0.246 to −0.115 across individuals, with all nine individuals negative), and also negative in C2 and C5, while C3 and C4 were close to zero. This is opposite in sign to the expectation under inbreeding and is not explained by the allele-frequency filter: recomputing FIS from the unfiltered call set changed it by at most 0.015 (C1, −0.164 unfiltered versus −0.179 filtered). We therefore base all statements about inbreeding on FROH and do not interpret the sign of FIS (Section 4.5).

Mutational load was likewise highest in the most inbred population: the nonsynonymous-to-synonymous minor-allele load ratio declined with decreasing altitude, from 1.34 in C1 to 1.15 in C4 (Figure S17), consistent with reduced efficiency of purifying selection in the small C1 population (FROH = 0.155).

## 4. Discussion

### 4.1. Differentiation Along the Altitudinal–Longitudinal Gradient

The central finding is that genomic differentiation between the four *L. sacra* populations is organised along a continuous altitudinal–longitudinal gradient rather than into four discrete units: PC1 ordered the populations along the transect, and the strongest axis of structure (K = 2) separated the high- from the mid-to-low-altitude populations—the two ends of a single ranked series rather than isolated clusters; because *L. wui* is represented by a single population, the species axis and the population axis cannot be separated, so we make no claim that altitude outranks species identity. Altitude and longitude are strongly correlated, so the gradient is best described as a composite axis, although geographic distance and altitudinal difference correlate at only 0.83 and are thus partly separable. In partial Mantel tests, altitudinal difference retained a significant association with differentiation after geographic distance was controlled, whereas geographic distance had no detectable effect once altitude was controlled (Section 3.2), pointing to the environmental component rather than isolation by distance. This inference rests on six population pairs and cannot identify which climatic variable is responsible because temperature, precipitation, ultraviolet radiation and atmospheric pressure all vary along the same axis. Comparable patterns are documented in other Qinghai–Tibet Plateau vertebrates [21], and in other lizards [24,25], providing a baseline for the delineation of evolutionarily significant units and conservation priorities in this species.

The diversity gradient supports this interpretation. Within *L. sacra*, π fell ~9-fold, and LD half-decay rose 27-fold across the transect, reflecting much smaller effective sizes at high altitude. These correlations are based on population means (*n* = 4), so they reflect a consistent rank order rather than an individual-level relationship. The ‘higher altitude, smaller effective size’ pattern may stem from lower productivity in high-altitude habitats; the higher diversity of C3/C4 is consistent with the lower gorges having acted as glacial refugia [4]. The negative Tajima’s D in C1 is not readily reconciled with the concurrent evidence for a small, highly inbred population, and because Tajima’s D was computed on MAF-filtered data (Section 2.4), we do not use it to infer the direction of demographic change. SMC++ shows persistently small C1/C2 sizes, and FROH rises ~25-fold from C3/C4 to C1. Critically, within *L. sacra* the altitudinal axis dominates the K = 2 solution, yet species-level divergence has accumulated far greater absolute differentiation (dXY = 0.639 vs. 0.142), indicating that intraspecific altitudinal differentiation remains shallow relative to the species boundary.

A further aspect of the gradient deserves comment. Serén et al. [25] found that allele-frequency change in the Tenerife lizard *Gallotia galloti* was not gradual but stepped, and our locus-level analysis points in the same direction: among the 3791 loci whose allele frequencies changed perfectly monotonically across the four *L. sacra* populations, the mean jump index was 0.60, against 0.48 for a linear cline simulated with the same endpoints and sampling noise and subjected to the same monotonicity filter (Kolmogorov–Smirnov *p* < 10^−300^), so a single altitudinal interval accounts for about 60% of the total change rather than the roughly one-third expected of a smooth cline. The population-level data agree: pairwise FST is flat among adjacent populations but rises steeply across longer separations, and a two-segment model fits FST/(1 − FST) against altitudinal difference better than a straight line (R^2^ 0.980 versus 0.918). Therefore, we describe the gradient as continuous in its population-level ordering but step-dominated at the level of individual loci. Three qualifications apply: neither analysis identifies the position of a threshold; the locus-level test compared the observed clines only against a strictly linear null and not against clines generated by drift, so it shows that they are steeper than a smooth gradient rather than adaptive; and with only six population pairs, the model comparison is suggestive rather than conclusive.

### 4.2. Species Status and Speciation Mode of L. wui

*L. wui* ranges from Pailong to the tropical rainforests of Mêdog [12]. Our Layue Hot Spring sample is the westernmost locality sampled in this study and lies at the upper altitudinal limit of the species, so the presence of only one *L. wui* population in our transect reflects where the transect was placed rather than a sampling shortfall. We therefore adopt a hierarchical framework: all population-level statements are made within *L. sacra* (four populations, 36 individuals) only, and the *L. wui* sample is used solely for species-level comparisons (delimitation, absolute divergence, gene-flow direction). To confirm robustness, we recomputed interspecific dXY after dropping each *L. sacra* population in turn; all four leave-one-out estimates (0.645–0.653) remained within 1% of the full-data value (0.646), indicating that a single *L. wui* population suffices to characterise interspecific divergence.

The species status of *L. wui* is supported by several independent lines of evidence: FST between C5 and each *L. sacra* population exceeded 0.80, C5 formed a long branch in the maximum-likelihood tree, and ADMIXTURE assigned it a distinct component at K = 5. We note that the FST contrast is the weakest of these lines of evidence, because the highest intraspecific value (0.675) approaches the lowest interspecific value (0.806). Because *L. wui* is represented by a single population, its grouping with C3/C4 at K = 2 reflects shared ancestral variation at the earliest-resolved axis rather than evidence about species identity. Two chromosome-scale *L. wui* genomes [13,14] provide a basis for integrating morphological, karyotypic and ecological evidence. Quantitatively, dXY was 4.8-fold higher between species than within *L. sacra* (0.136), with 35.0% fixed SNPs (Figure S18).

Both the shared ancestral components with C3/C4 at K = 2 and the topology (C5 sister to C4 in the IQ-TREE tree, to C3 in TreeMix) suggest *L. wui* derived from a mid-to-low-altitude ancestor shared with eastern *L. sacra*—peripatric speciation driven by geographic and ecological isolation in hot-humid gorge habitats [49]. The TreeMix edge from C1 to C3 (weight = 0.444) suggests historical gene flow, though its direction cannot be established; it did not cross the species boundary, and the lack of recent gene flow corroborates reproductive isolation, as is known in other QTP lizards and plants [50,51].

Two features of the inferred topologies require comment. First, in the maximum-likelihood tree, C5 (*L. wui*) is sister to C4, rendering *L. sacra* paraphyletic with respect to *L. wui*. A peripatric origin of *L. wui* from an ancestor shared with eastern *L. sacra* is the most parsimonious reading, but we cannot exclude that C4 is a distinct unit, or that the pattern partly reflects retained ancestral polymorphism; discriminating between these alternatives will require sampling between Mainling and Pailong and the analysis of additional nuclear and mitochondrial data. Second, the maximum-likelihood tree placed C5 as sister to C4 whereas TreeMix placed it as sister to C3; with a single *L. wui* population, neither topology should be treated as decisive. Therefore, we base species-level statements on the convergence of FST, dXY and the proportion of fixed differences rather than on any single topology.

### 4.3. Candidate Genes for Altitudinal and Species-Level Differentiation

Four of the thirteen candidate genes (*SHROOM4*, *HDAC8*, *BMP15*, *DGKD*) cluster in a ~115 kb interval on scaffold 26 and show altitudinal clines stronger than the genome-wide background (Figure S19), suggesting a potentially important genomic region associated with altitudinal differentiation; however, evidence from haplotype structure and linkage disequilibrium is required to establish whether these genes represent a co-adapted unit.

The candidates span functional classes consistent with roles in high-altitude physiology: cytoskeletal remodelling (*FLNC*, *SHROOM4*; cf. [15,52]), epigenetic regulation (*HDAC8*; [53]), lipid/energy signalling (*DGKD*; [54]), glycolysis (*Eno3*, *PKM*, *HK2*), and oxygen sensing (*CYBA*, *Piezo1*) [55,56]. Several candidates derive from the interspecific C1 vs. C5 comparison and may, therefore, reflect species-level as well as altitudinal differentiation; because drift in small populations can also produce extreme values, functional validation is required to confirm adaptive significance.

The wound-healing and monosaccharide-transport genes in C3 vs. C4 may reflect adaptation to low temperature and strong UV at high altitude; the marginal carbonate-dehydratase enrichment in C2 vs. C3 (*p*.adjust = 0.055) suggests a possible role in respiratory gas exchange and acid–base balance.

### 4.4. Conservation Genomic Implications

Our findings have direct conservation implications. C1 (Nyemo, 3758 m), the westernmost and highest-altitude *L. sacra* population, had the lowest diversity and smallest effective size, and carries ~278 Mb (15.5%) of its genome in ROH (FROH = 0.155), including long 2–4 Mb segments indicative of recent inbreeding—an order of magnitude above C3/C4. Under the SSP585 scenario, C1 shows the largest projected genetic offset (Figure 5)—a relative ranking of genomic vulnerability rather than a direct measure of extinction risk—and its elevated nonsynonymous load (1.34) indicates reduced purifying selection [48]. As the western range limit of the species, C1 is a key sentinel population [57] and a priority for conservation.

Although *L. wui* (C5) had the highest diversity here, it is only the westernmost population sampled here, at the upper altitudinal limit of the species (2311 m), and the species’ structure along the low-altitude Pailong–Mêdog reach remains unknown. Given the very limited recent gene flow, we recommend treating each population as an independent management unit and prioritising C1 and the unsampled low-altitude *L. wui* populations.

### 4.5. Limitations and Prospects

Seven design limits warrant comment. First, all reads were mapped to a single *L. sacra* reference; the two species are strongly divergent (dXY between species = 0.646 at variable sites; Section 2.9), which may bias against the more divergent *L. wui*, and a dual-reference or pangenome approach would recover species-specific variation more completely. Second, the most recent and deepest epochs of coalescent Ne inference are weakly constrained, so absolute divergence times should be interpreted with caution. Third, the partial Mantel tests indicate which axis carries the signal but cannot quantify the relative contributions of environmental and geographic isolation because only six population pairs are available and the axes are correlated at 0.83. Fourth, variant filtering used VQSR on a cohort of 44 individuals, which is smaller than the sample sizes for which the algorithm was designed; although the recommended tranches were applied, a systematic comparison against hard-filtered call sets would be desirable. Fifth, FIS is negative in C1 and C2, opposite in sign to the expectation under inbreeding and to FROH; the cause is most likely technical (for example, genotype-calling or variant-ascertainment effects) but cannot be established, so we rest all statements about inbreeding on FROH alone. Sixth, Tajima’s D is strongly sensitive to the allele-frequency filter (recomputed values differ by up to 0.91 depending on the filter), so we report it descriptively and do not use it to infer demographic change. Seventh, the SMC++ effective sizes depend on the assumed mutation rate, and the value used here may be several-fold too large, so the reported Ne (for example ≈164 for C1) may be correspondingly too small; this is consistent with the nucleotide-diversity and LD-based estimates, which are one to two orders of magnitude higher. Therefore, we report the ordering of populations rather than absolute values. Extending sampling through the unsampled Pailong–Mêdog gorge would complete the cline and, by adding pairs at similar altitudes, allow the two components to be separated; transcriptomic and phenotypic data would further validate the candidate genes.

## 5. Conclusions

In conclusion, the deeply incised Yarlung Zangbo valley functions as a one-dimensional geographic template along which genomic divergence is ordered continuously at the population level: within *L. sacra*, the altitudinal–longitudinal axis is the dominant axis of structure, and the gradients in diversity, LD, effective size and inbreeding all track it, consistent with putative incipient differentiation along the gradient, in which intraspecific altitudinal divergence (dXY = 0.142) remains well below the species axis (0.639). Nevertheless, *L. sacra* and *L. wui* represent two independently evolving species, with *L. wui* likely derived from a mid-to-low-altitude ancestor shared with *L. sacra* via peripatric speciation.

Whether local adaptation has progressed to the point of reducing gene flow is untested here and would require common-garden or reciprocal-transplant data; candidate genes for high-altitude adaptation (*FLNC*, *SHROOM4*, *HDAC8*, *Eno3*, *PKM*, *HK2*, *CYBA*, *Piezo1*) likewise warrant functional validation. Comparable transects in other incised valleys are needed to test whether this coincidence is general.

## Figures and Tables

**Table 1:** Genetic diversity parameters of each population. Ho, observed heterozygosity; He, expected heterozygosity; FIS, inbreeding coefficient (negative values indicate heterozygote excess relative to Hardy–Weinberg expectation). π is expressed per site, its denominator being the window length in bp, whereas Ho, He and FIS are expressed per variant site and were computed on the MAF-filtered variant set; the two conventions differ by the density of retained variants (about 4.8 × 10^−4^ per bp in C1), which is why π is some three orders of magnitude smaller than He. FIS is reported for completeness only; because it is conditional on the same filtering, FROH (Section 3.9) is used as the primary measure of inbreeding.

Population	Species	Altitude (m)	π	Ho	He	FIS	Tajima’s D
C1	*L. sacra*	3758	0.00015	0.368	0.312	−0.181	−0.303
C2	*L. sacra*	3582	0.0004	0.380	0.354	−0.076	0.731
C3	*L. sacra*	3265	0.0011	0.359	0.359	0.000	1.127
C4	*L. sacra*	3017	0.0013	0.384	0.385	0.003	1.179
C5	*L. wui*	2311	0.0015	0.417	0.404	−0.032	1.206

## Data Availability

The whole-genome resequencing data (raw FASTQ files) for all 44 individuals have been deposited in the NCBI Sequence Read Archive under BioProject PRJNA1524104 (SRA study SRP734744) and are publicly available at https://www.ncbi.nlm.nih.gov/bioproject/PRJNA1524104 (accessed on 30 October 2026).

## Supplementary Materials

The following supporting information can be downloaded at: https://www.mdpi.com/article/10.3390/biology15211982/s1, Figure S1: Maximum-likelihood phylogenetic tree; Figure S2: Maximum-likelihood population tree and gene flow inferred by TreeMix; Figure S3: SMC++ reconstruction of effective population size; Figure S4: Genomic inbreeding coefficient (FROH); Figure S5: Cross-validation (CV) error as a function of K in ADMIXTURE; Figure S6: Heatmap of pairwise FST; Figure S7: Boxplots of genetic diversity parameters; Figure S8: Genetic diversity indices versus altitude; Figure S9: Linkage disequilibrium decay; Figure S10: Residual fit from TreeMix model; Figure S11: Genome-wide FST between C1 and C5; Figure S12: Genome-wide log_10_ π ratio between C1 and C5; Figure S13: Joint FST and π-ratio analysis for C1 vs C5; Figure S14: Cumulative number of runs of homozygosity; Figure S15: Environmental principal component analysis; Figure S16: Scaffold-wise distribution of the 3791 perfectly monotonic altitudinal cline candidates; Figure S17: Relative mutational load; Figure S18: Interspecific versus intraspecific divergence; Figure S19: Anchoring candidate genes to altitudinal clines. Table S1: Per-individual runs-of-homozygosity (ROH) statistics; Table S2: Pairwise genetic differentiation (FST) among populations; Table S3: Linkage disequilibrium decay statistics.
