# Supplementary material for: Genomic Differentiation Along an Altitudinal–Longitudinal Gradient in Yarlung Zangbo Rock Agamas (Laudakia)
Source: Biology (Basel). 2026 Nov 2;15(21):1982. doi: 10.3390/biology15211982 (PMC13762386; doi:10.3390/biology15211982)
Supplement: Supplementary file 1 [file biology-15-01982-s001.zip › biology-4638887-supplementary.pdf]

Supplementary Materials

Genomic differentiation along an altitudinal–longitudinal gradient in Yarlung Zangbo rock agamas (*Laudakia*)

Supplementary figures and tables

Supplementary Figures

Figure S1: Maximum-likelihood phylogenetic tree of 44 individuals based on LD-pruned genome-wide SNPs (IQ-TREE, GTR model, 1,000 UFBoot replicates). The circular tree shows the topology of the five populations (C1–C5); colours denote populations as in the legend.

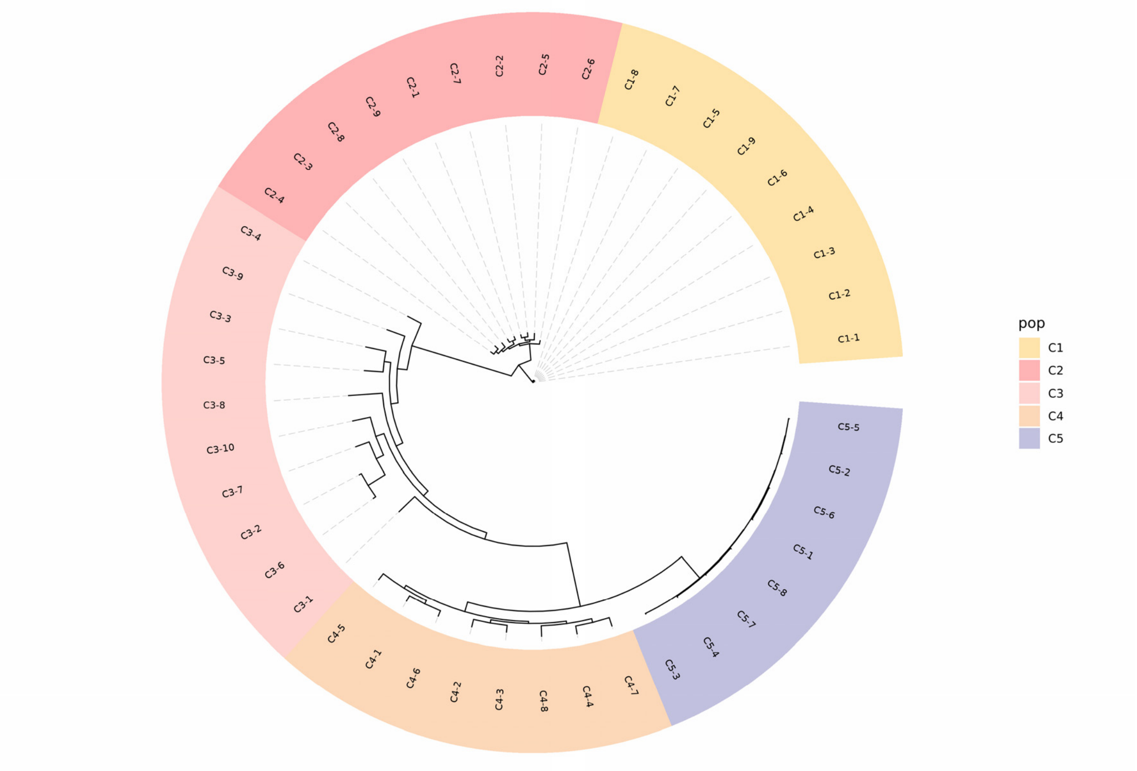

Figure S2: Maximum-likelihood population tree and gene flow inferred by TreeMix (k = 1,000, m = 1). The arrow indicates a migration edge from the C1 lineage to the C3 lineage; colour denotes migration weight. The x-axis is the drift parameter.

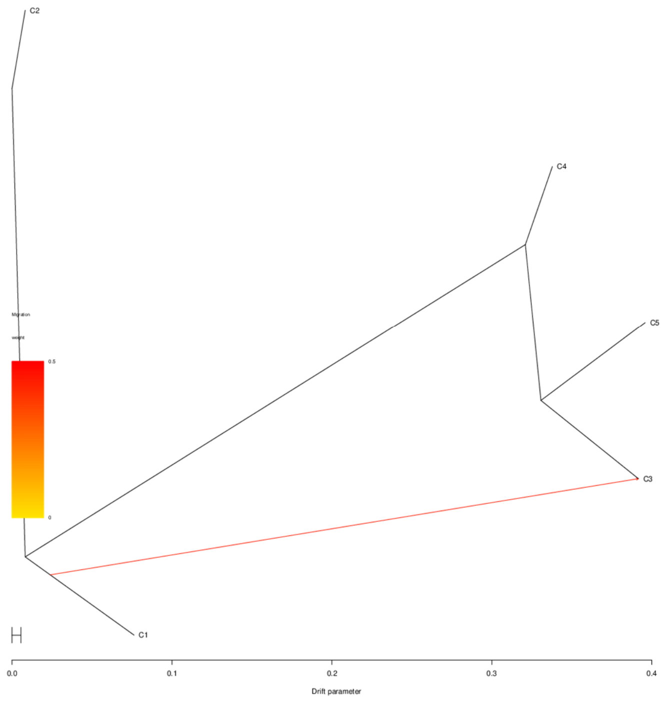

Figure S3: SMC++ reconstruction of effective population size (Ne) through time for the five populations (C1–C5), assuming a generation time of 3 years. The x-axis is years before present (log scale) and the y-axis is Ne (log scale); colours denote populations as in the legend. The high-altitude populations C1/C2 show persistently small Ne, whereas the mid-altitude C3 maintains the largest Ne.

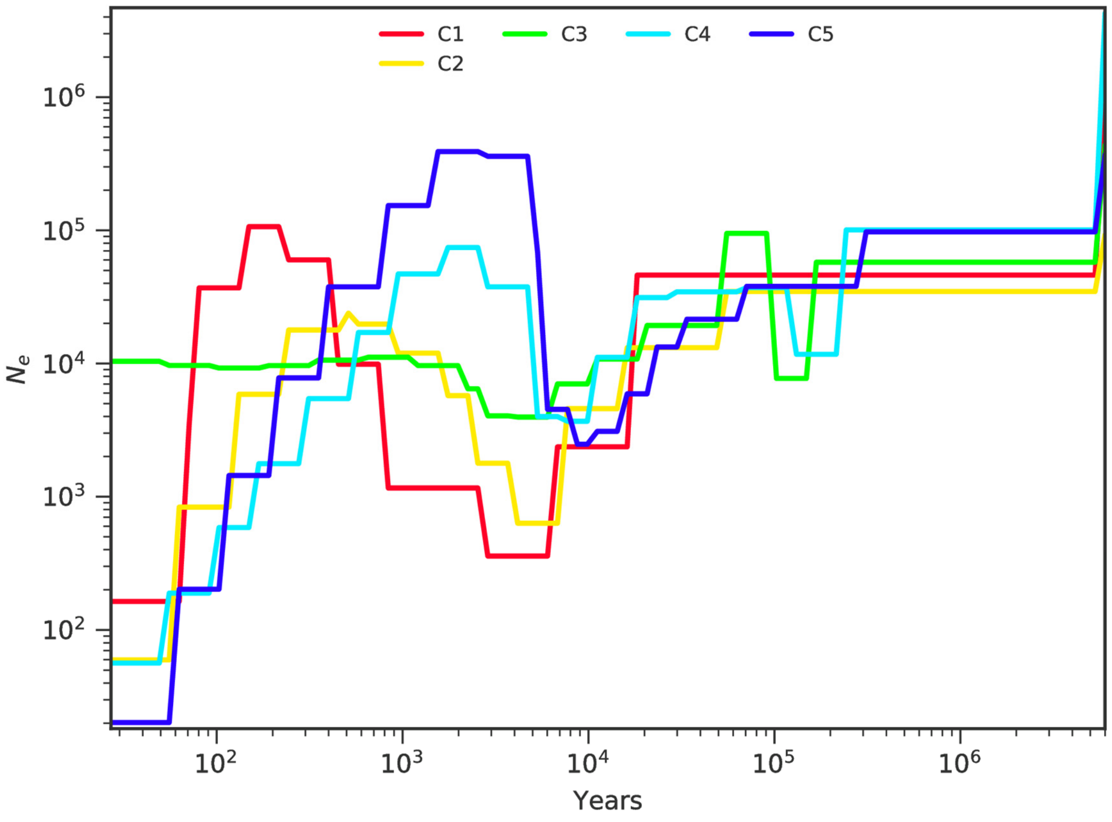

Figure S4: Genomic inbreeding coefficient (FROH) across the five populations (C1–C5), shown as violin plots with embedded box plots. FROH increases steeply with altitude within *L. sacra*, peaking in the highest population C1, and is lowest in *L. wui* (C5).

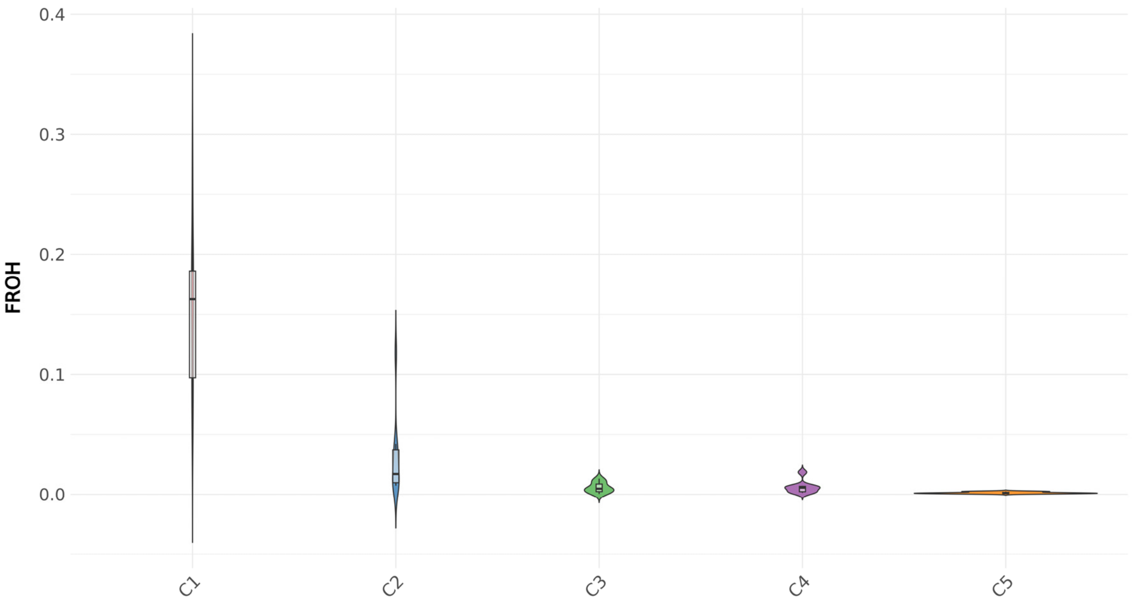

Figure S5: Cross-validation (CV) error as a function of K (K = 1–10) in ADMIXTURE analysis. The CV error reaches its minimum at K = 5 (highlighted), indicating the optimal number of ancestral clusters.

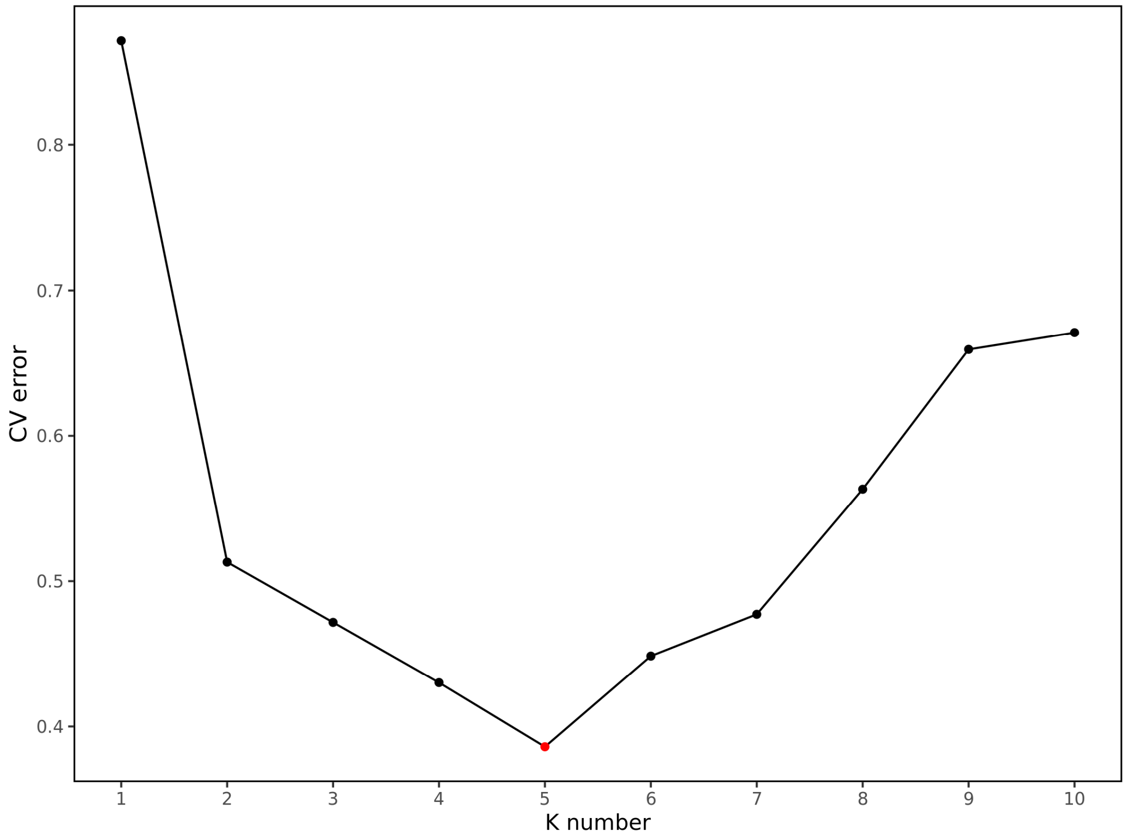

Figure S6: Heatmap of pairwise FST among the five *Laudakia* populations (lower-triangle matrix; rows C2–C5, columns C1–C4). Cell values give the FST for each population pair and colour encodes the magnitude.

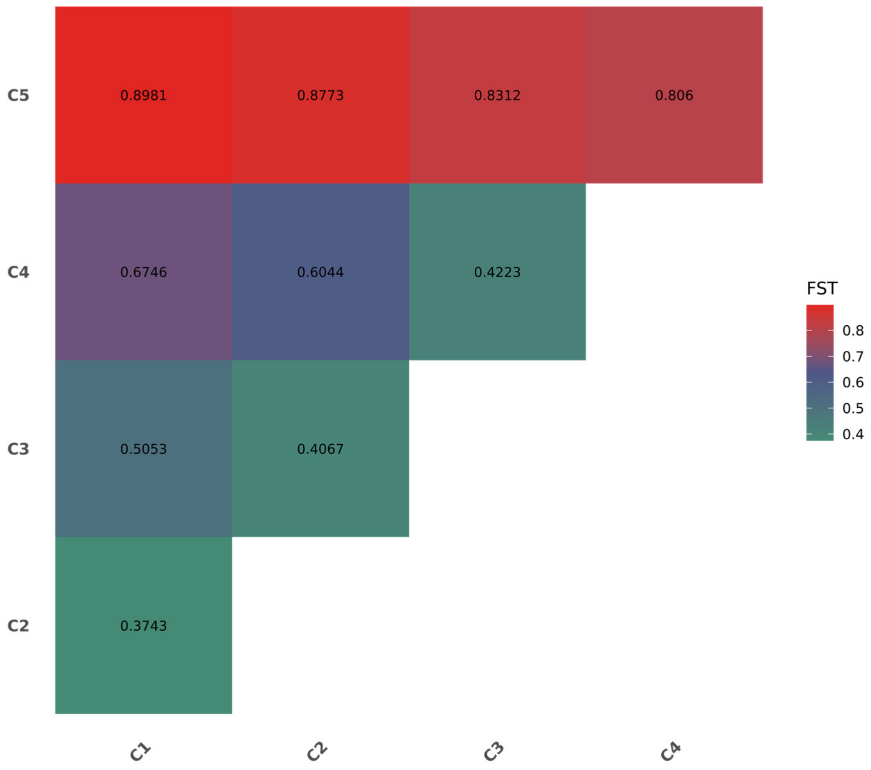

Figure S7: Boxplots of genetic diversity parameters across the five populations (C1–C5): (a) nucleotide diversity (π), (b) SNP density (variants/kb), (c) observed heterozygosity (Ho), (d) expected heterozygosity (He), (e) inbreeding coefficient (F) and (f) Tajima's D. Box colours denote populations as in the legend.

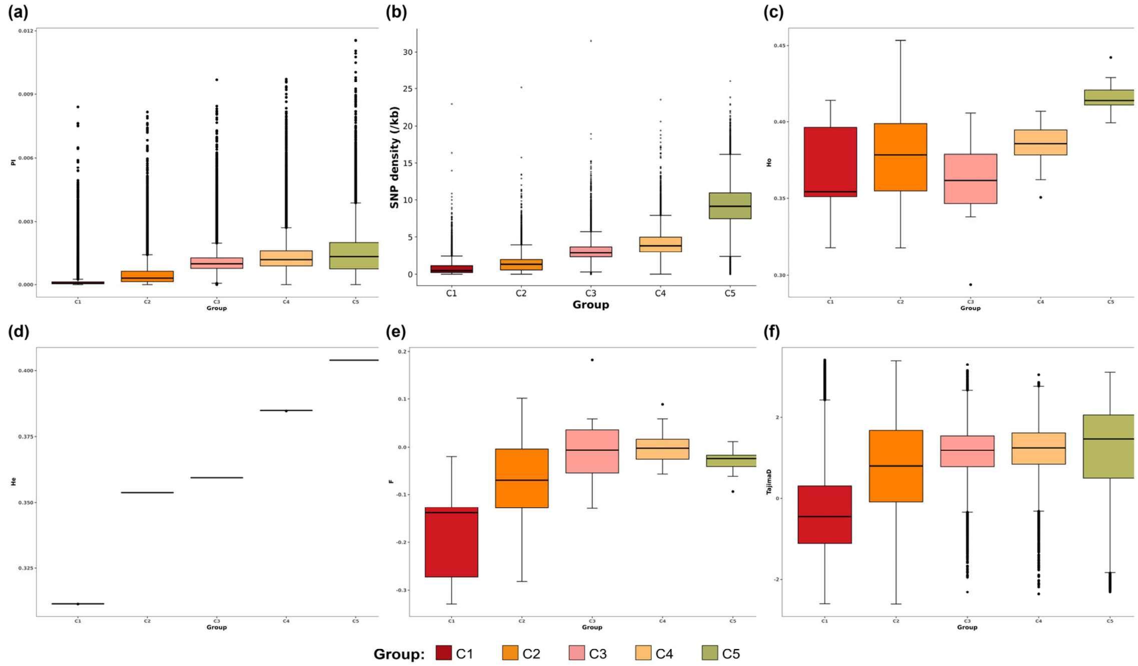

Figure S8: Genetic diversity indices versus altitude. Panels A–D show nucleotide diversity (π), expected heterozygosity (He), SNP density and LD half-decay distance against sampling-site altitude. Blue circles, *L. sacra* (C1–C4) with the dashed regression line fitted on these four populations; red diamond, *L. wui* (C5), plotted separately and excluded from regression. Each panel reports Spearman ρ and the linear-regression r and P values.

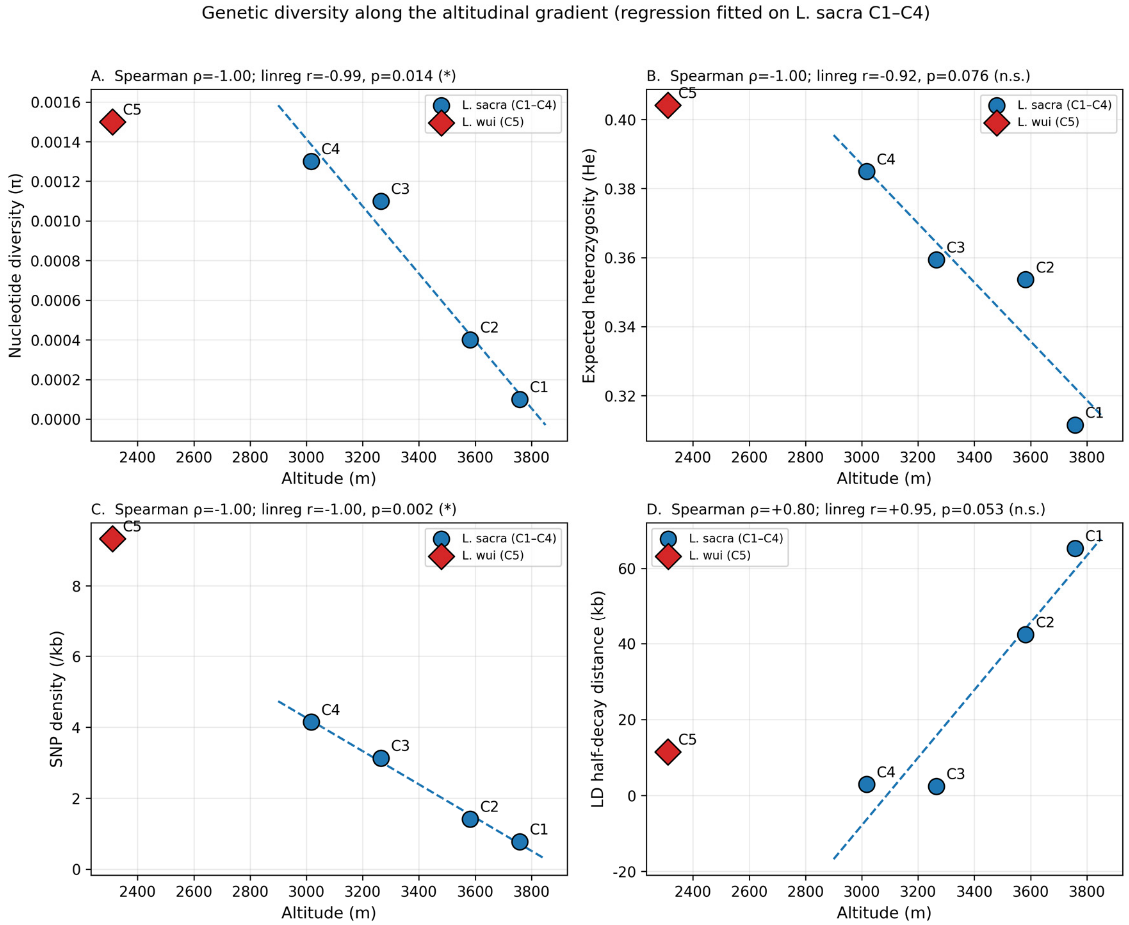

Figure S9: Linkage disequilibrium decay curves for the five populations.

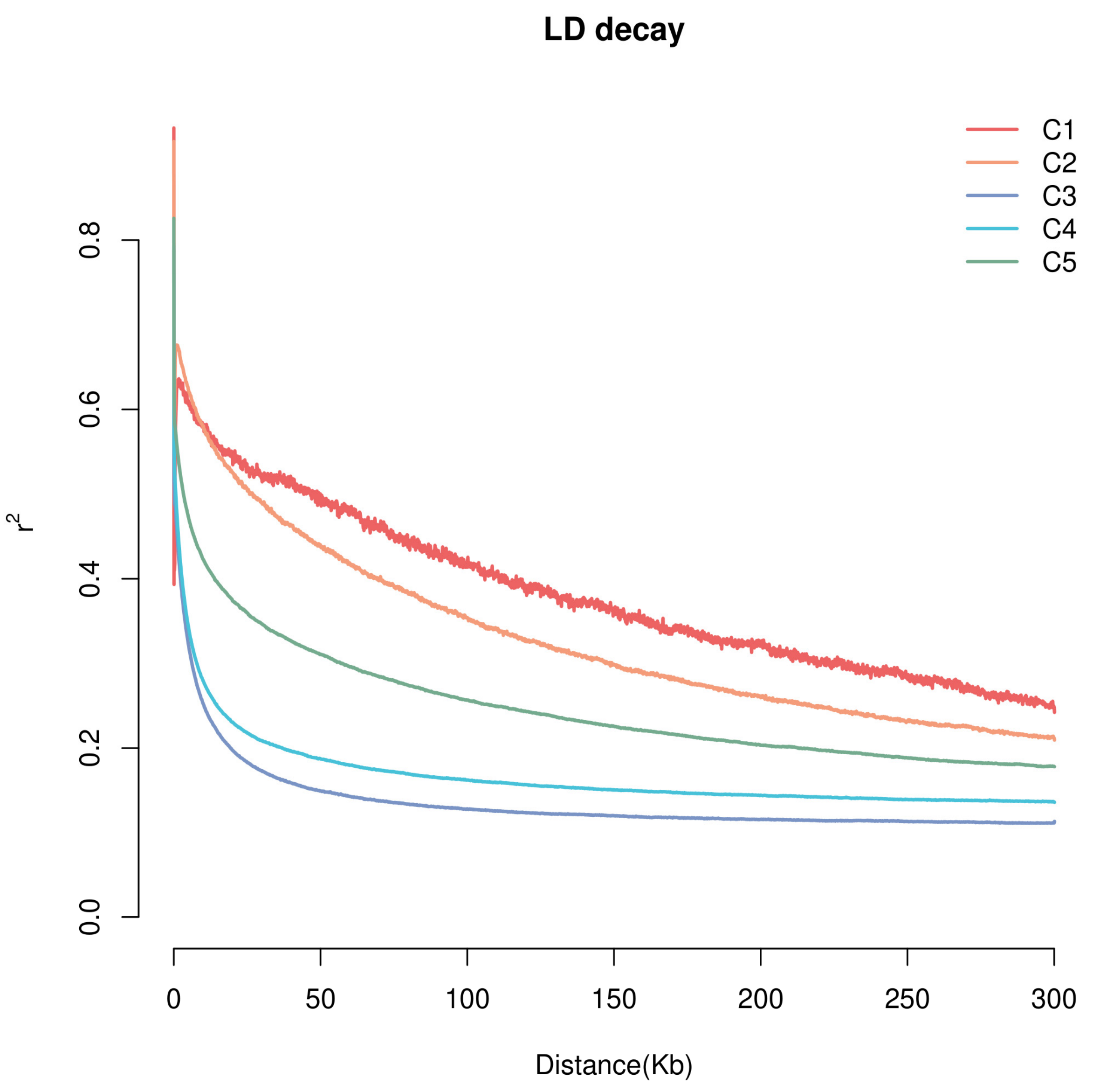

Figure S10: Residual fit from TreeMix model (k = 1000, m = 1).

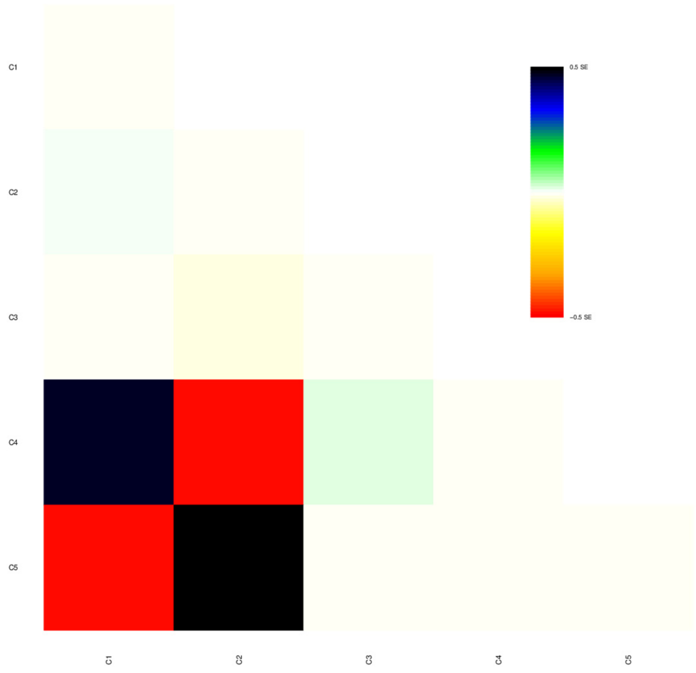

Figure S11: Genome-wide FST between C1 and C5 (100 kb windows). Red line: top 5% threshold.

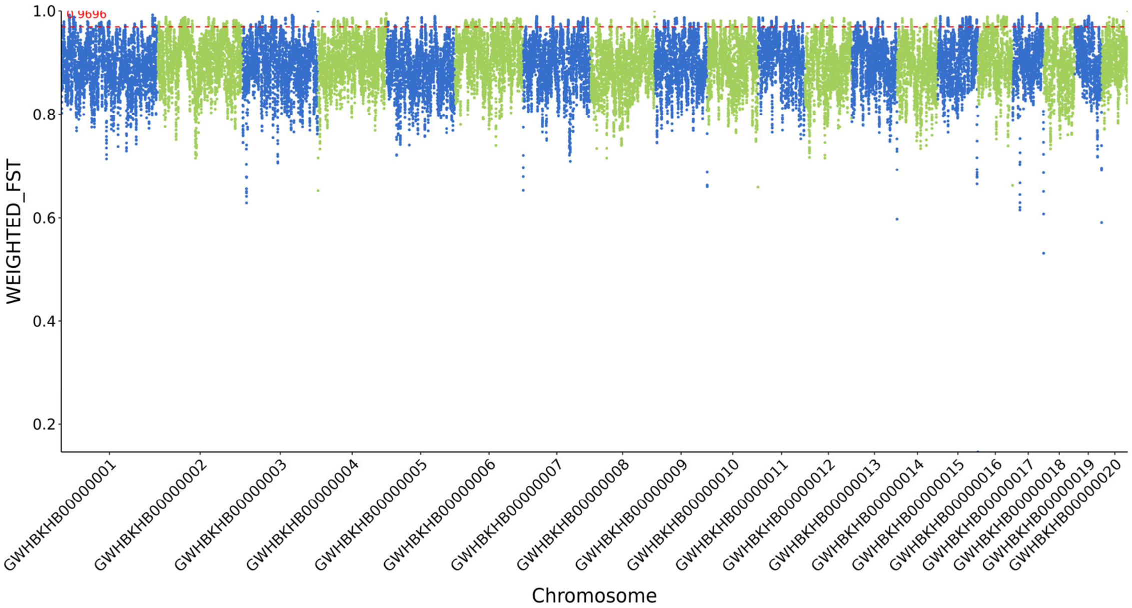

Figure S12: Genome-wide $\log_{10} \pi$ ratio between C1 and C5 (100 kb windows). Red dashed lines indicate the upper (top 5%) and lower (bottom 5%) thresholds.

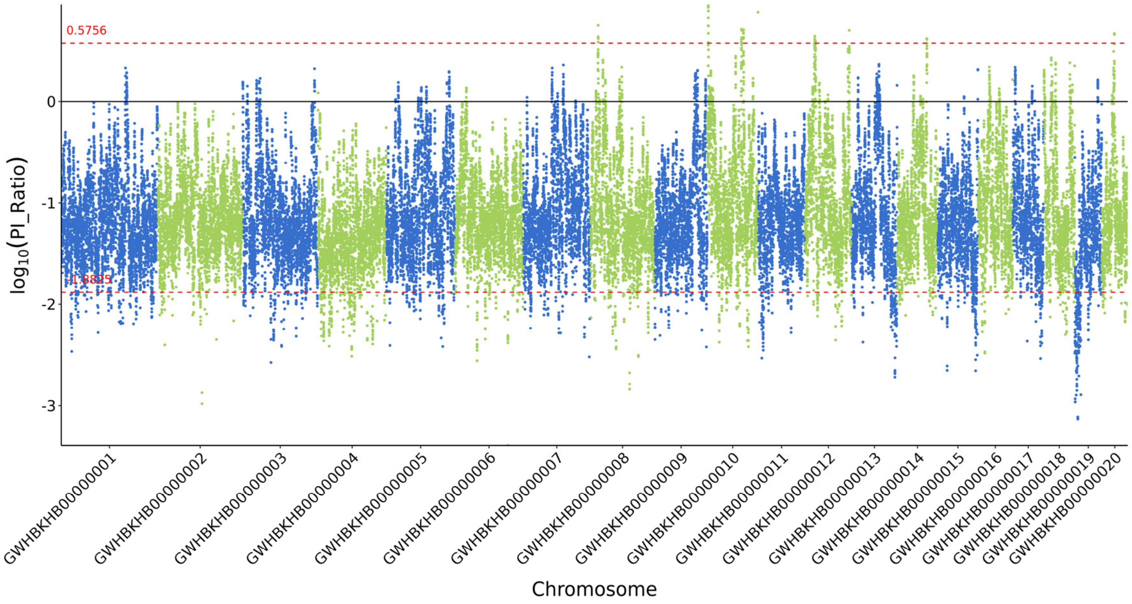

Figure S13: Joint FST and π-ratio analysis for C1 vs C5. Purple: C1 candidates; pink: C5 candidates.

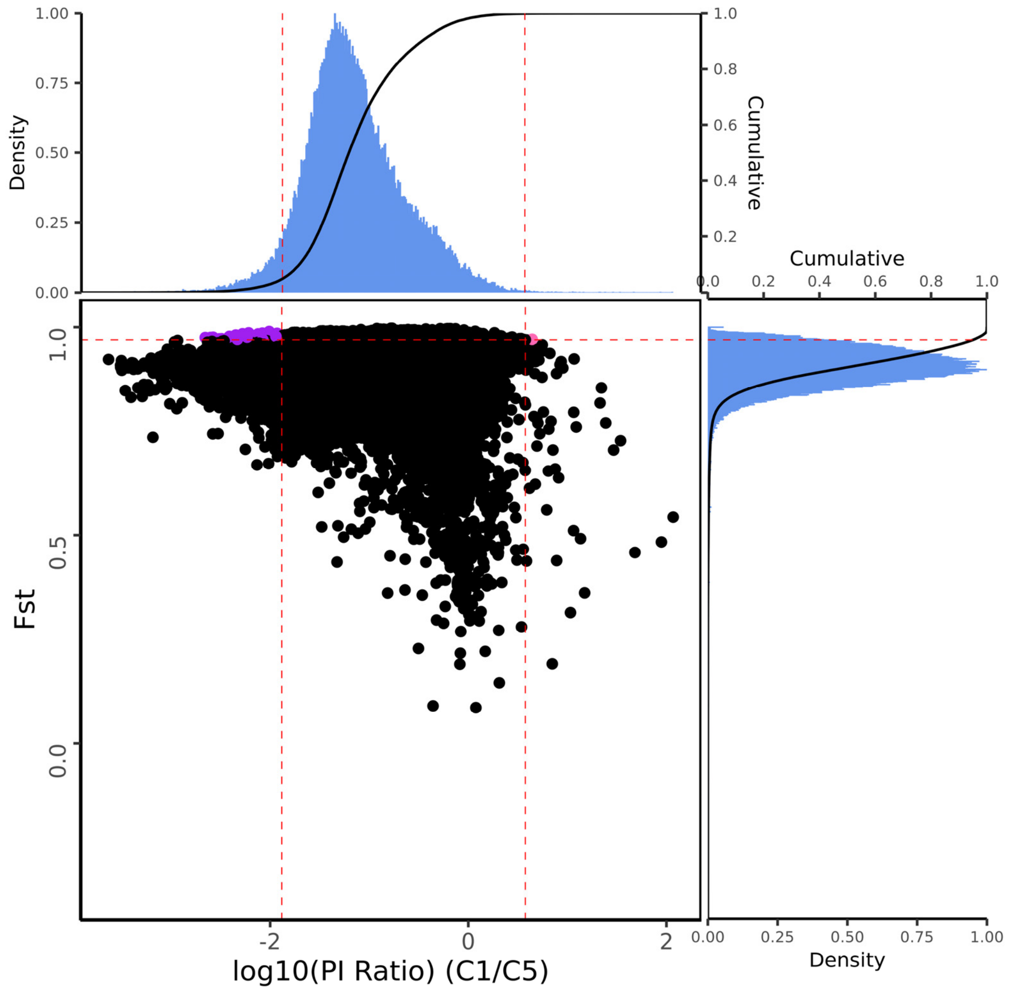

Figure S14: Cumulative number of runs of homozygosity (ROH) per individual by length class (0.5–1, 1–2 and 2–4 Mb) for the five populations (C1–C5). The high-altitude population C1 carries the most abundant and the longest ROH segments.

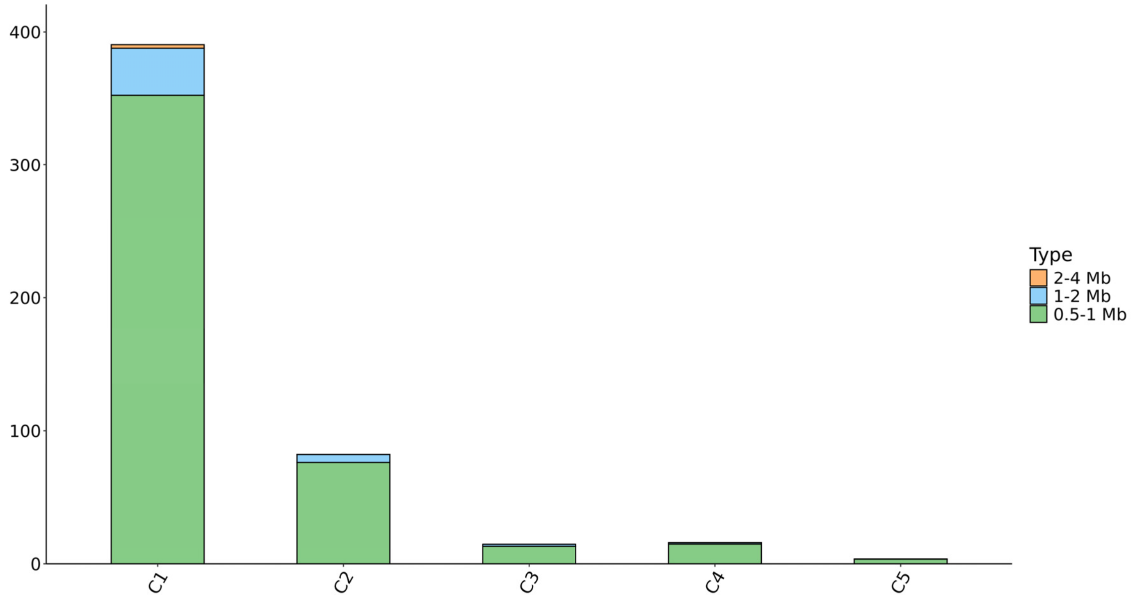

Figure S15: Environmental principal component analysis (PCA) of nine climatic and topographic variables at the five sampling sites. Variables were derived from standard physical relationships (moist adiabatic lapse rate = 6.5 degC/km; oxygen partial pressure from the barometric formula; orographic precipitation gradients along the Yarlung Zangbo Valley) rather than from direct climatic measurements. (A) Scree plot: PC1 captures 98.2% of total variance. (B) PC1 loadings. (C) Elevation vs PC1 score (r = -0.996). Population colours: red = C1, orange = C2, green = C3, blue = C4, purple = C5.

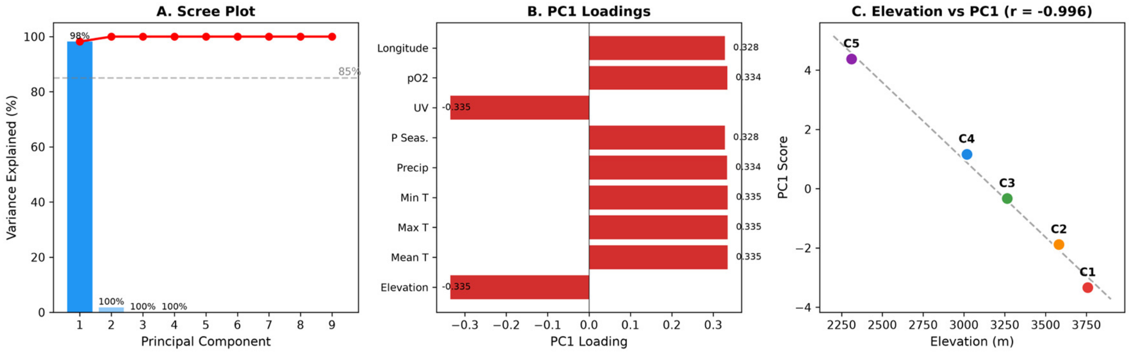

Figure S16: Scaffold-wise distribution of the 3,791 perfectly monotonic altitudinal cline candidates ($|\rho| = 1$) across the 226 SNP-bearing scaffolds of the *L. sacra* assembly (GWHBKHB00000000; 284 scaffolds). Each point is one scaffold, plotted as the observed candidate count against the count expected under the genome-wide rate (1.91%); the dashed diagonal marks the expectation. The candidates are distributed close to the genome-wide expectation, consistent with a polygenic rather than a region-concentrated architecture of altitudinal differentiation.

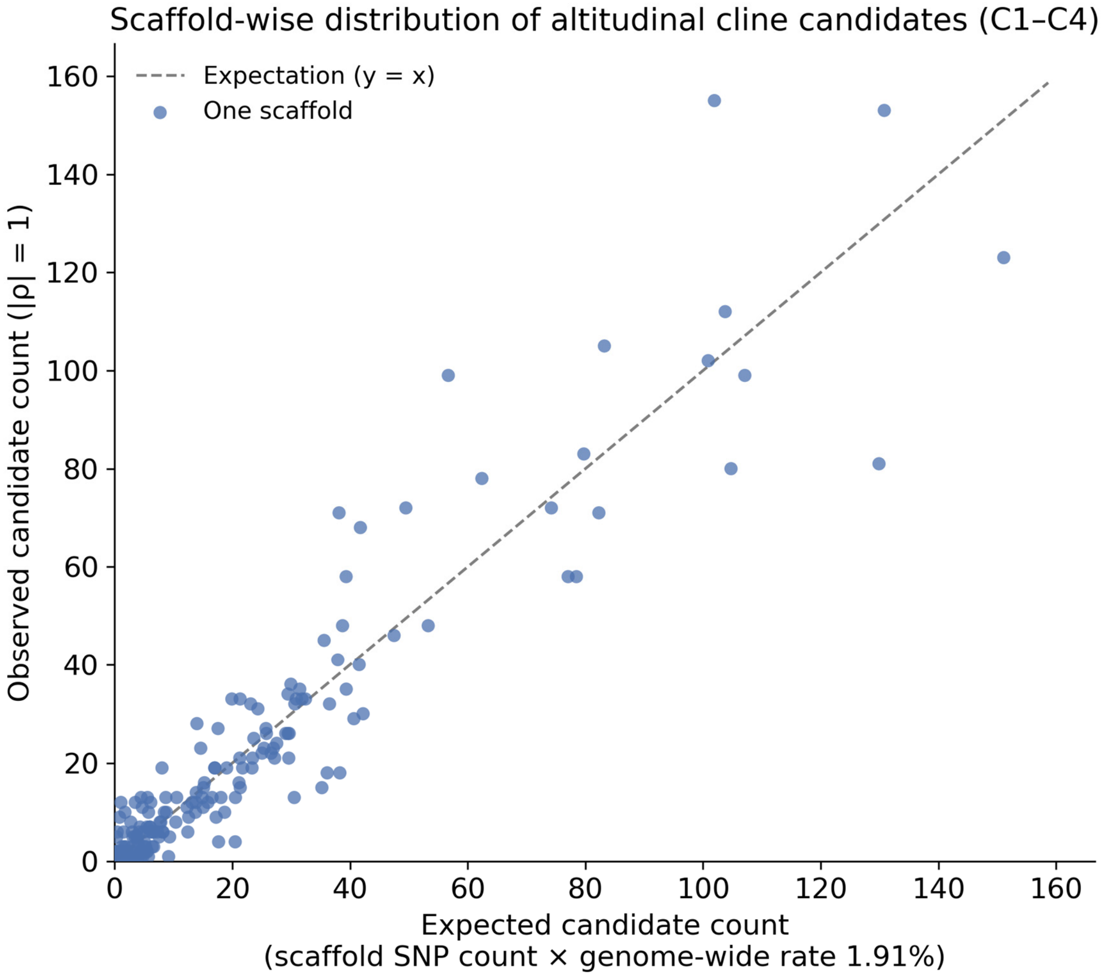

Figure S17: Relative mutational load along the altitudinal gradient: (A) Nonsynonymous-to-synonymous ratio for each of the four *L. sacra* populations; the dashed line indicates the neutral expectation (=1). (B) Relative load increases monotonically with altitude.

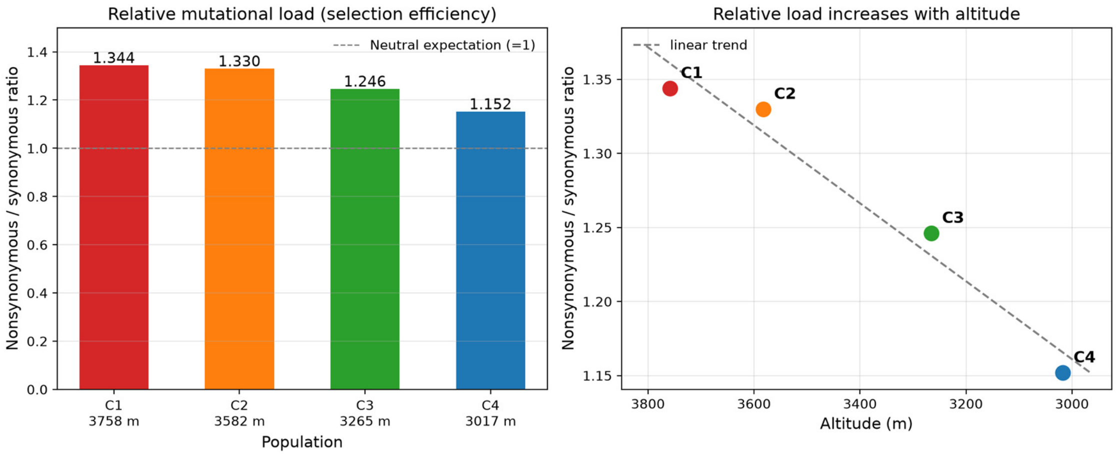

Figure S18: Interspecific versus intraspecific divergence: (A) dXY (probability that two alleles drawn at random from the two groups differ), (B) proportion of fixed-difference SNPs, and (C) Hudson's FST, comparing C5 (*L. wui*) versus C1–C4 (*L. sacra*) (interspecific, red) with C1–C4 pairwise (intraspecific, blue).

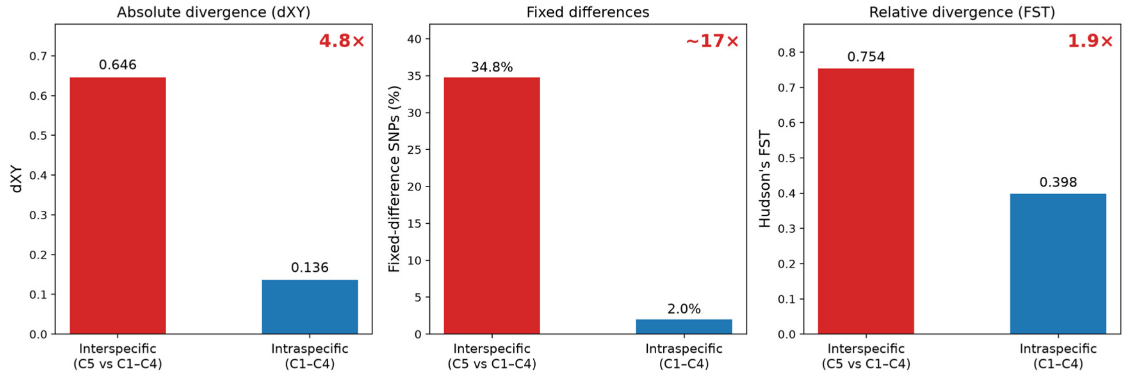

Figure S19: Anchoring candidate genes to altitudinal clines: (A) Boxplot of the absolute Spearman correlation (|ρ|) with altitude for genome-wide background SNPs (random sample, n = 3,000) versus candidate-gene SNPs (n = 1,457). Candidate-gene clines are significantly stronger (Mann–Whitney U test, P < 0.001). (B) Mean |ρ| for each of the 13 candidate genes (FLNC, SHROOM4, HDAC8, DGKD, BMP15, Eno3, PKM, HK2, CYBA, Piezo1, MTHFD2, ARRB2 and SLC4A5), coloured by functional class; the dashed line indicates the genome-wide mean (0.29).

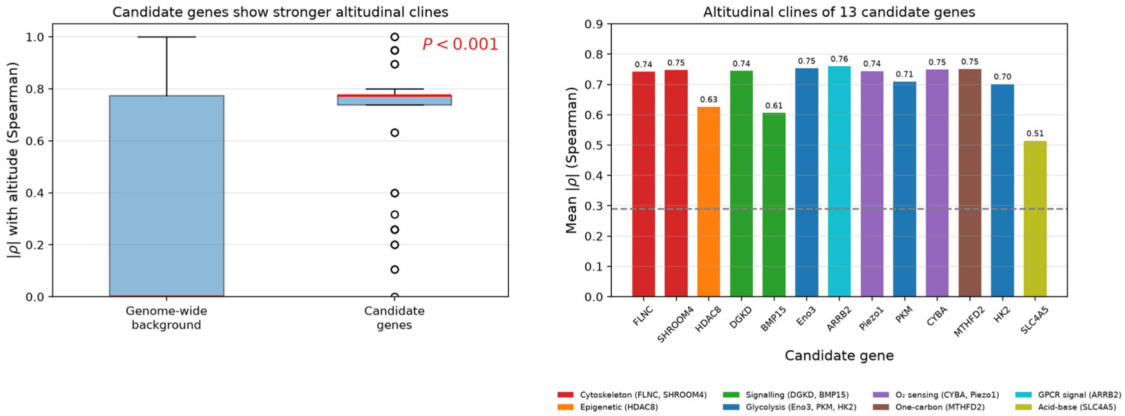

Supplementary Tables

Table S1: Per-individual runs-of-homozygosity (ROH) statistics and genomic inbreeding coefficient (FROH) for all 44 individuals across the five populations (C1–C5). Sum ROH is the total length of ROH per individual; N ROH is the number of ROH segments; FROH = Sum ROH / autosomal genome length (1,797,983,225 bp). Population means are given in bold.

Population	Individual	Altitude (m)	Sum ROH (Mb)	N ROH	FROH
C1	C1-1	3,758	352.5	496	0.1960
C1	C1-2	3,758	320.7	455	0.1784
C1	C1-3	3,758	334.7	486	0.1862
C1	C1-4	3,758	272.2	388	0.1514
C1	C1-5	3,758	488.7	661	0.2718
C1	C1-6	3,758	292.5	412	0.1627
C1	C1-7	3,758	129.8	183	0.0722
C1	C1-8	3,758	137.8	190	0.0767
C1	C1-9	3,758	174.8	244	0.0972
C1	Mean	3,758	278.2	391	0.1547
C2	C2-1	3,582	66.3	97	0.0369
C2	C2-2	3,582	13.6	22	0.0076
C2	C2-3	3,582	30.5	43	0.0169
C2	C2-4	3,582	21.3	32	0.0118
C2	C2-5	3,582	17.5	24	0.0097
C2	C2-6	3,582	13.0	18	0.0072
C2	C2-7	3,582	75.1	111	0.0418
C2	C2-8	3,582	53.2	74	0.0296
C2	C2-9	3,582	212.2	317	0.1180
C2	Mean	3,582	55.9	82	0.0311
C3	C3-1	3,265	23.6	33	0.0131
C3	C3-2	3,265	9.0	14	0.0050
C3	C3-3	3,265	17.3	25	0.0096
C3	C3-4	3,265	8.0	13	0.0045

C3	C3-5	3,265	10.1	15	0.0056
C3	C3-6	3,265	2.7	5	0.0015
C3	C3-7	3,265	7.2	9	0.0040
C3	C3-8	3,265	19.7	27	0.0110
C3	C3-9	3,265	0.6	1	0.0004
C3	C3-10	3,265	3.1	4	0.0017
C3	Mean	3,265	10.1	15	0.0056
C4	C4-1	3,017	3.1	5	0.0017
C4	C4-2	3,017	2.3	3	0.0013
C4	C4-3	3,017	33.2	46	0.0184
C4	C4-4	3,017	4.3	6	0.0024
C4	C4-5	3,017	12.5	15	0.0069
C4	C4-6	3,017	11.8	19	0.0066
C4	C4-7	3,017	10.6	17	0.0059
C4	C4-8	3,017	9.5	16	0.0053
C4	Mean	3,017	10.9	16	0.0061
C5	C5-1	2,311	1.8	2	0.0010
C5	C5-2	2,311	0.9	1	0.0005
C5	C5-3	2,311	1.6	3	0.0009
C5	C5-4	2,311	1.2	2	0.0007
C5	C5-5	2,311	2.3	3	0.0013
C5	C5-6	2,311	4.5	6	0.0025
C5	C5-7	2,311	2.7	4	0.0015
C5	C5-8	2,311	4.3	7	0.0024
C5	Mean	2,311	2.4	4	0.0013

Table S2: Pairwise genetic differentiation (FST) among populations

	**C1**	**C2**	**C3**	**C4**	**C5**
**C1**	—	0.374	0.505	0.675	0.898

**C2**		—	0.407	0.604	0.877
**C3**			—	0.422	0.831
**C4**				—	0.806
**C5**					—

Table S3: Linkage disequilibrium decay statistics

**Population**	**Half-decay distance (kb)**	**Half-decay r^2**	**Max r^2**
**C1**	65.3	0.466	0.932
**C2**	42.4	0.458	0.917
**C3**	2.4	0.394	0.789
**C4**	2.9	0.396	0.793
**C5**	11.5	0.413	0.826
